# Hematopoietic effects of Fufang E’jiao Jiang revealed by microbiome, metabolome and transcriptome analyses: a multi-omics strategy

**DOI:** 10.3389/fimmu.2025.1561477

**Published:** 2025-06-12

**Authors:** Yueting Mo, Xiyuan He, Peixin Shi, Yifei Ning, Mingmei Zhou, Hao Cui, Ting Zhang

**Affiliations:** ^1^ Institute for Interdisciplinary Integrative Medicine Research, Shanghai University of Traditional Chinese Medicine, Shanghai, China; ^2^ Faculty of Life Science and Technology, Kunming University of Science and Technology, Kunming, China; ^3^ Laboratory of Sustainable Utilization of Panax Notoginseng Resources, State Administration of Traditional Chinese Medicine, Kunming, China

**Keywords:** Fufang E’jiao Jiang, anemia, microbiome, metabolome, transcriptome

## Abstract

**Introduction:**

Fufang E'jiao Jiang has been extensively utilized to replenish qi and nourish blood as the homology of medicine and food.

**Methods:**

We analyzed the effects of FEJ on cyclophosphamide and acetylphenylhydrazine-induced anemia mice through gut microbiome analysis, fecal metabolomics, and transcriptome sequencing.

**Results:**

FEJ markedly alleviated the anemia symptoms in the mice. FEJ markedly alleviated the anemia symptoms caused by cyclophosphamide and acetylphenylhydrazine induction. FEJ improved the gut microbiome imbalance by inhibiting the proliferation of harmful bacteria *Turicibacter, Akkermansia* and *Tuzzerella*. Fecal metabolomic data showed that FEJ regulated metabolic disorders in anemia mice and was probably associated with L-leucine, L-proline, glycine, phenylalanine, propanoic acid and butanoic acid. Transcriptome analysis indicated the amelioration of anemia was predominantly associated with the hematopoietic cell lineage, osteoclast formation and B cell receptor signaling pathway. According to Spearman's correlation analysis, there was a strong link between gut microbiota and hematopoietic index, metabolites and genes.

**Discussion:**

Our study supports the application of FEJ in anemia treatment.

## Introduction

Anemia is a prevalent disease seen in clinical settings that is defined by a reduction in red blood cells (RBC) and hemoglobin concentrations. It impacted up to one-third of the world’s population (1). In traditional Chinese medicine theory, anemia is defined by blood deficiency and represents a condition of organ malnutrition and blood dysfunction. Patients or animals suffering from anemia frequently face impaired hematopoietic function, peripheral blood pancytopenia, diminished internal organ function, malnutrition, or myelosuppression during severe illnesses (2). In clinical, two primary strategies are employed to treat anemia: stimulating the production of RBC and providing more iron to the bone marrow. However, these methods are accompanied by negative effects. Severe cases may require blood transfusions, which could potentially result in hemolysis or heart failure (3). Accordingly, the development of safe and efficacious strategies for treating anemia is a concern in the medical community.

Fufang E’jiao Jiang (FEJ) is a syrup made from *Asini Corii Colla* and four food-medicine homology ingredients *Ginseng Radix et Rhizoma Rubra* (*Panax ginseng* C.A.Mey.), *Codonopsis Radix* (*Codonopsis pilosula* Nannf.), *Crataegi Fructus* (*Crataegus pinnatifida* Bunge.) and *Rehmanniae Radix Praeparata* (*Rehmannia glutinosa* (Gaertn.) Libosch. ex DC.) using modern processing and refining technology (4–9). Complex chemical composition of FEJ showed common challenges, including ambiguous bioactive components and unidentified therapeutic targets. A total of 72 chemical ingredients in FEJ were identified by HPLC–MS, including organic acids, flavonoids, phenylethanoid glycosides, notoginsenosides and ginsenosides (10). Oligosaccharides, which have significant immune-boosting effects, were key constituents of FEJ, and 13 oligosaccharides identified via ultra-performance liquid chromatograms combined with triple quadrupole mass spectrometry (UPLC-QQQ-MS) (6). FEJ is produced exclusively by Shandong Dong-E-E-Jiao Co., Ltd in Dong’e, China. Its sales surpassed one billion Yuan in China in 2012. The efficacy of FEJ is replenishing qi and nourishing blood (8) and it has been clinically employed in the adjuvant treatment of anemia by way of blood supplementation (6). In a myelosuppression mouse model, treatment with FEJ promoted the restoration of bone marrow hemopoietic activity by improving bone marrow hematopoietic niche, preventing bone marrow nucleated cells (BMNCs) from apoptotic cell death and stimulating the expressions of cytokines essential to hematopoiesis (8). The percentage of hematopoietic stem cells and the quantities of burst-forming unit-erythroid (BFU-E) and colony-forming unit granulocyte-monocyte (CFU-GM) in BMNCs of mice with myelosuppressive were significantly increased by FEJ supplementation (11). Nonetheless, a systematic investigation of the underlying molecular mechanism of FEJ’s hematopoietic effects has not yet be conducted.

In light of the prominent efficacy of FEJ in the treatment of anemia, this study aimed to elucidate the potential mechanism of FEJ in alleviating anemia in mice induced by cyclophosphamide (CTX) and acetylphenylhydrazine (APH), with an emphasis on the role of gut microbiota, metabolites, and genes. The ability of FEJ to ameliorate hematopoietic dysfunction was assessed, and pathological analyses of bone and spleen tissues were performed. Following the analysis of gut microbiota and fecal metabolites from fecal samples, we proceeded to perform gene expression profiling of the spleen. The findings of this study offered insights into the pharmacological mechanisms of FEJ in regulating hematopoiesis.

## Materials and methods

### Material preparation

FEJ (No. 2208021) was provided by Shandong Dong-E-E-Jiao Co., Ltd (Dong’e, China). The above plant names have been checked with http://mpns.kew.org and http://www.worldfloraonline.org/taxon/wfo-0000263606. Accessed on: 10 Jan 2025’. CTX and APH were obtained from Aladdin Biochemical Technology Co., Ltd (Shanghai, China). The ELISA kits for erythropoietin (EPO) were acquired from Enzyme-linked Biotechnology Co., Ltd (Shanghai, China). Kits for Malondialdehyde (MDA), superoxide dismutase (SOD), total antioxidant capacity (T-AOC) and bicinchonininc acid (BCA) were provided by Nanjing Jiancheng Bioengineering Institute (Nanjing, China).

Twenty-four Kunming (KM) mice, consisting of equal numbers of males and females, and weighing 18–22 g, were obtained from Beijing Vital River Laboratory Animal Technology Co., Ltd. (Beijing, China). The mice were maintained under standard conditions with unlimited access to food and water (22°C ± 1°C temperature and 12 h light/dark cycle) to allow acclimation to the laboratory environment. The study received approval of the Animal Studies Ethics Committee of Shanghai University of Traditional Chinese Medicine (Ethical number: PZSHUTCM2302110002).

### Animal experiment

#### Experimental design (Animal modeling and feeding)

**Figure 1:**
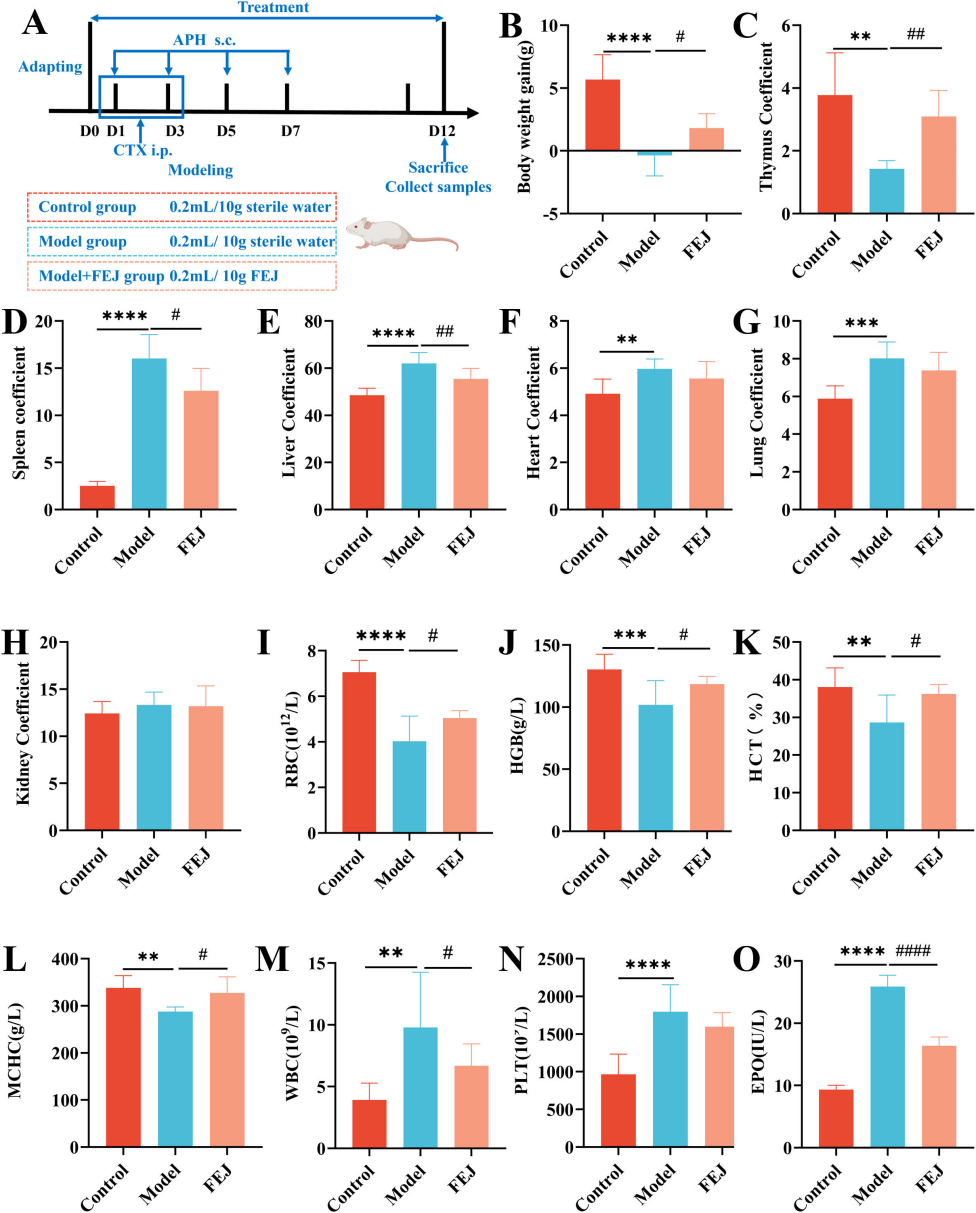
Impact of FEJ on weight loss, organ injury, blood routine and hematopoiesis cytokine abnormalities in mice. **(A)** The process of the experiment. **(B)** Body weight gain. **(C)** Thymus coefficient. **(D)** Spleen coefficient. **(E)** Liver coefficient. **(F)** Heart coefficient. **(G)** Lung coefficient. **(H)** Kidney coefficient. **(I)** The level of RBC. **(J)** The level of HBG. **(K)** The level of HCT. **(L)** The level of MCHC. **(M)** The level of WBC. **(N)** The level of PLT. **(O)** The level of EPO. Values are presented as mean ± SD (n = 8). ^**^ *p* < 0.01, ^***^ *p*<0.001 and ^****^ *p* < 0.0001 versus control group. ^#^ *p* < 0.05, ^##^ *p*< 0.01 and ^####^ *p* < 0.0001 versus model group.

The mice were evenly distributed into three groups: the control group, model group and FEJ group, with 8 mice in each group (**Figure 1A**). The model and FEJ groups, but not the control group, received subcutaneous injections of APH at a dose of 20 mg/kg on the first, third, fifth and seventh days, and intraperitoneal injections of CTX at a dose of 40 mg/kg on the first to third days to induce anemia (12). Subsequently, the control group and model group were gavaged with water, whereas the FEJ group was gavaged with FEJ (8 mL/kg) last 12 days. FEJ is particularly popular for its remarkable performance in tonifying blood. In our previous study, we utilized it as a positive control and discovered that an 8 mL/kg dose of FEJ significantly alleviated anemia (13, 14). Consequently, this dosage was still used in this study. After the final administration, mice were positioned in a sterile frame for fecal collection, and a minimum of three fresh feces particles were gathered for subsequent analysis. The researcher collected blood from the orbital sinus and reserved if for biochemical assays. The mice were anesthesia with ether and euthanized by cervical dislocation. All mice were dissected and thymus, spleen, liver, heart, lung, and kidney tissues were washed in 0.9% normal saline and weighed. The femur and portions of the spleen were preserved in 4% paraformaldehyde. All other samples were quickly transferred in −80°C for later use.

#### Body weight gain and organ indexes

Body weight gain was calculated using the formula: body weight gain = the final weight (12th day of treatment) (g)- the initial weight (g). The organ coefficient was computed using the formula: organ coefficient (mg/g)=organ wet weight (mg)/body weight (g) (15).

#### Routine blood test

The blood samples were obtained in tubes containing EDTA-K_2_ anticoagulant. The white blood cells (WBC), RBC, hemoglobin (HGB) and platelets (PLT) were quantified in mice using Mindray automatic veterinary blood cell analyzer (BC-30Vet) (16). The remaining blood samples were utilized for additional testing.

#### Measurements of hematopoiesis related cytokine in serum

To isolate the supernatant, blood samples were centrifuged at 3000 rpm for 10 minutes at 4°C (5415R, Eppendorf, Hamburg, Germany). The serum was then analyzed for EPO levels using enzyme-linked immunoassays at 450 nm, following the manufacturer’s instructions (17).

#### Assessing oxidative stress factors in the liver

The liver tissue was homogenized and the T-AOC, SOD, and MDA levels were analyzed using a commercial kit, following the manufacturer’s instructions (18).

#### Bone histological changes

After decalcification, the femur was processed into paraffin-embedded thin slices. These thin slices were stained with hematoxylin-eosin (H&E) for the examination of the histopathological changes within the bone marrow (15).

#### Spleen histological changes

The spleen was embedded in paraffin and thin slices of the paraffin blocks were stained with H&E for the histological examination (19). Additionally, paraffin spleen sections were employed for immunohistochemical staining (20). The spleen sections were trans-sectioned through incubation with 3% hydrogen peroxide in methanol for 25 min, incubated with primary antibodies overnight, incubated with a horseradish peroxidase-conjugated secondary antibody, and stained with peroxidase substrate DAB kit. Subsequently, sections were counterstained with hematoxylin before examination under light microscopy. We analyzed the percentages of positive staining areas using the ImageJ 2.15.1 software (https://imagej.net/software/fiji/).

#### Measurement of short-chain fatty acids levels

The levels of SCFAs in feces were determined using a method adapted from a previous study (21). Briefly, a mixed standard stock solution A was prepared in n-butanol, comprising acetic acid, propionic acid, butyric acid, isobutyric acid, valeric acid, isovaleric acid, hexanoic acid and isohexanoic acid (Sigma Chemical Co., St. Louis, MO, USA). Stock solution B was prepared with n-butanol and internal standard 2-ethylbutyric acid (Sigma Chemical Co., St. Louis, MO, USA). Standard A and B solutions were mixed and diluted with n-butanol into 7 gradient concentrations of solutions. Then, feces (20 mg) were mixed with 500 μL 0.5% phosphoric acid water, ground and centrifuged at 13000 g for 15 min (Centrifuge 5430R, Eppendorf, Hamburg, Germany). The supernatant (200 μL) was combined with 200 μL of a 10 μg/mL 2-ethylbutyric acid solution in n-butanol, and then centrifuged at 13000 g for 5 min. Samples were then analyzed using a GC system (8890B-5977B GC/MSD, Agilent Technologies, CA, USA) fitted with an HP-FFAP column (30 m × 0.32 mm × 0.25 µm, Agilent, CA, USA). The operating conditions were as follows: nitrogen as the carrier gas at a flow rate of 1mL/min. The temperature was initially set to 80°C, then ramped up to 120°C at a rate of 20°C/min, followed by an increase to 160°C at a rate of 5°C/min, and held at 220°C for 3 minutes. The concentrations of SCFAs were calculated using linear regression standard curve.

#### Microbiome analysis via 16S rRNA sequencing

The total bacterial DNA was extracted from the fecal samples using the E.Z.N.A.^®^ soil DNA Kit (Omega Bio-tek, Norcross, GA, U.S.). The V3-V4 hypervariable region of the 16S rRNA gene was targeted for amplification with primer pairs 338F (ACTCCTACGGGAGGCAGCAG) and 806R(GGACTACHVGGGTWTCTAAT) (22), and performed on an ABI GeneAmp^®^ 9700 PCR thermocycler (ABI, CA, USA). Sequencing was utilized on the Illumina MiSeq PE300 platform (Illumina, San Diego, USA). UPARSE 7.1 (23) clustered the optimized sequences into operational taxonomic units (OTUs) at a 97% similarity level. Bioinformatic analysis was conducted on the Majorbio Cloud platform (https://cloud.majorbio.com). Non-metric multidimensional scaling analysis (NMDS) was conducted to analyze differences in bacterial community composition at the OTU level. A Linear Discriminant Analysis (LDA) score greater than 3.5 was considered the distinguishing feature. Each group comprised six samples.

#### Metabolomics analysis

The procedure for extracting fecal metabolites involved the following steps: Weighing 100 mg of feces, mixing with 400 µL of a 1:1 methanol/water solution, homogenizing using a Tissue Grinder (Tiss-24, Jingxin Industrial Development Co., Shanghai, China) and subsequently centrifuging for 10 min at 4 °C and 13000rpm. 300 µL mixture was added 10 µL of heptadecanoic acid methanol solution, dried under nitrogen and combined with 50 µL of methoxyl amine hydrochloride pyridine solution for 90 minutes (24). After the reaction, 50 µL BSTFA was added and the mixture was subjected to a silicon alkylation reaction at 70°C for 1 hour. Finally, the supernatant was collected for analysis.

Metabolites analysis was performed using an Agilent GC-MS system equipped with an Agilent J&W DB-5ms Ultra Inert column (30 m × 0.25 mm × 0.25 µm). Nitrogen served as the carrier gas at a flow rate of 1 mL/min. The injection volume was 1 µL. The temperature program began at 80°C, then ramped up to 120°C at a rate of 20°C/min, followed by a further increase to 160°C at a rate of 5°C/min, and held at 220°C for 3 minutes.

Experimental data were analyzed using the metaboanalyst 6.0 online platform. Statistical analyses, including Student’s t-test and multivariate analysis of variance, were conducted. Metabolites with a VIP score > 1 and a *p* -value < 0.05 were considered significantly differentially expressed.

#### Gene expression profiling analysis

Spleen tissue RNA was extracted using the MJZol total RNA extraction kit (Majorbio, Shanghai, China) following the provided guidelines. The messenger RNA was then isolated through polyA selection with oligo(dT) beads and fragmented. Next, double-stranded cDNA was synthesized with the SuperScript double-stranded cDNA synthesis kit (Invitrogen, CA) employing random hexamer primers. The cDNA was processed for end-repair, phosphorylation and adapter addition in line with library construction protocol. Libraries were selected for 300 bp fragments on 2% Low Range Ultra Agarose and then PCR amplified with Phusion DNA polymerase (NEB) for 15 cycles. Libraries were quantified with Qubit 4.0 and sequenced on the NovaSeq X Plus platform(PE150). Reads underwent trimming and quality control using fastp (25) and were aligned to reference genome HISAT2 software (26). StringTie was employed to assemble reads in a reference-guided manner (27). DEGs (differential expression genes) were identified by the transcripts per million reads (TPM) expression analysis, with RSEM for gene quantification (28). DESeq2 was applied for differential expression analysis (29), identifying DEGs with |log2FC|≧1 and FDR<0.05 as significantly differentially expressed. KEGG pathway analysis was performed using Python scipy software at Bonferroni-corrected *p*-value < 0.05.

#### Statistical analysis

We used GraphPad prism 8.3.0 software for statistical analysis. Results were expressed as mean ± SD, and statistical significance was set at *p* < 0.05.

## Results

### Impact of FEJ on body weight, organ indexes, peripheral blood cells and EPO

The model group exhibited a significant reduction in body weight gain and thymus coefficient compared to the control group (*p*< 0.01, **Figures 1B, C**), and a significant increase in the coefficients of spleen, liver, heart and lung (*p*< 0.01, **Figures 1D–G**, **Supplementary Figure S1**). FEJ treatment showed a trend towards enhancing body weight gain and thymus coefficient and improving the swelling of spleen and liver (*p* < 0.05). However, FEJ had no significant effects on the heart and lung coefficient (*p* < 0.05, **Figure 1H**). No significant difference in the kidney coefficient was observed among the control, model and FEJ groups.

After 12 days treatment with FEJ, we determined the levels of blood routine indicators (**Figures 1I–N**). The model group showed significantly decreased levels of RBC, HGB, HCT and MCH (*p* < 0.01), and significantly increased levels of WBC and PLT (*p* < 0.01) compared to the control group, confirming the successful establishment of the anemic mouse model. Treatment with FEJ significantly elevated the RBC, HGB, HCT and MCHC (*p*< 0.05) and reduced the WBC (*p*< 0.01). Similarly, ELISA kits were used to detect the level of EPO in serum. FEJ significantly ameliorated the increase of EPO level in the model group (*p*<0.0001, **Figure 1O**).

### Impact of FEJ on liver oxidative stress markers

**Figure 2:**
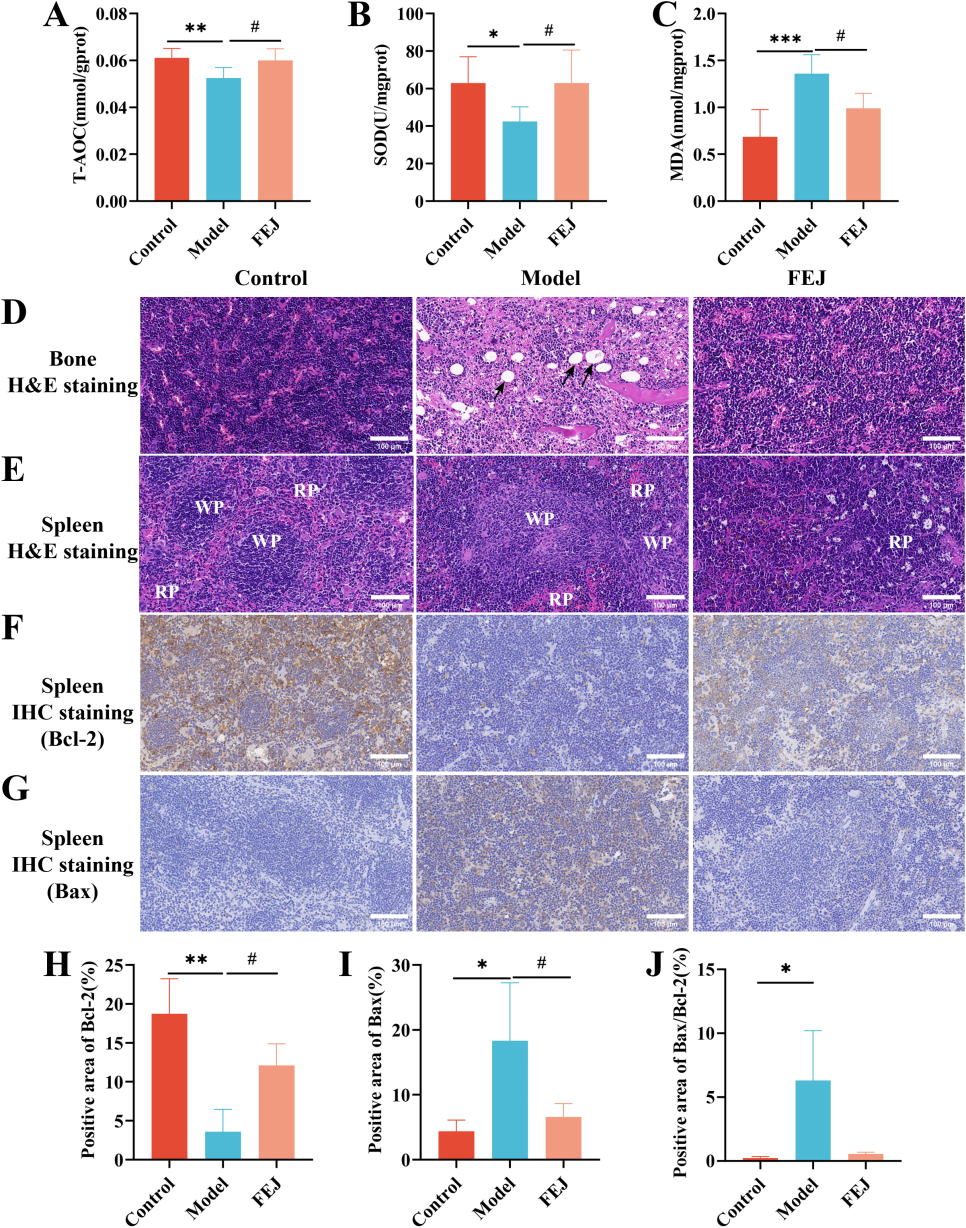
FEJ alleviated liver oxidative stress and promoted the recovery of bone and spleen tissue damage. The level of T-AOC **(A)**, SOD **(B)** and MDA **(C)** in liver. Values are presented as mean ± SD (n = 6). Pathological sections of bone marrow **(D)** and spleen **(E)**. (400×, scale bar: 100 μm). Immunohistochemical staining for Bcl-2 **(F)** and Bax **(G)** of spleen. (400×, scale bar: 100 μm). The percentages of positive staining area of immunohistochemical staining for Bcl-2 **(H)**, Bax **(I)** and Bax/Bcl-2 **(J)**. The black arrow indicates myeloid cell adipocytosis in **(D)**. In **(E)** red pulp is denoted by RP, white pulp is denoted by WP. Values are presented as mean ± SD (n = 3). ^*^ *p* < 0.05, ^**^ *p* < 0.01 and ^***^ *p* < 0.001 versus control group. ^#^ *p* < 0.05 versus model group.

T-AOC, SOD and MDA levels in the liver serve as oxidative stress indicators. The control and FEJ groups demonstrated significantly higher levels of T-AOC and SOD compared to the model group (**Figures 2A, B**). On the other hand, the MDA content was significantly elevated in the model group compared to the control and FEJ groups (**Figure 2C**).

### Impact of FEJ on bone histological features

The bone marrow is crucial for hematopoiesis in mice, serving as active hematopoietic tissue (30), and the impact of FEJ on bone marrow histopathology was assessed using H&E staining. Compared with the control group, the model group exhibited a significant abnormal proliferation and severe fatty degeneration of the bone marrow cells, leading to increased adipocyte formation and a subsequent decrease in erythroid, granulocytic and megakaryocytic cell counts. However, the FEJ group displayed an elevated bone marrow cellular density with plenty of hematopoietic cells, and there was a significant decrease in the number of adipocytes (**Figure 2D**).

### Impact of FEJ on spleen histological features

The spleen is also an active hematopoietic tissue (30). H&E staining revealed distinct red pulp (RP) and white pulp (WP) in the spleen of the control group, with clear and obvious boundaries, and an apparent splenic trabecular structure. In contrast, the RP and WP were not clearly defined, and the WP was atrophied in the model group. FEJ treatment led to a clearer boundary between the RP and WP and an expansion of the WP’s marginal area (**Figure 2E**). Bcl-2 and Bax are important proteins in regulating cell apoptosis. We observed that the expression of Bcl-2 decreased significantly (*p* < 0.01), the expression of Bax increased significantly(*p* < 0.05) and the ratio of Bax to Bcl-2 increased (*p* < 0.05) in the model group. Although FEJ administration was not able to restore the ratio of Bax to Bcl-2(*p* > 0.05), it significantly enhanced the expression of Bcl-2 (*p* < 0.05) and significantly reduced the expression of Bax significantly (*p* < 0.05) compared to the model group (**Figures 2F–J**).

### Impact of FEJ on the SCFAs in the feces

**Figure 3:**
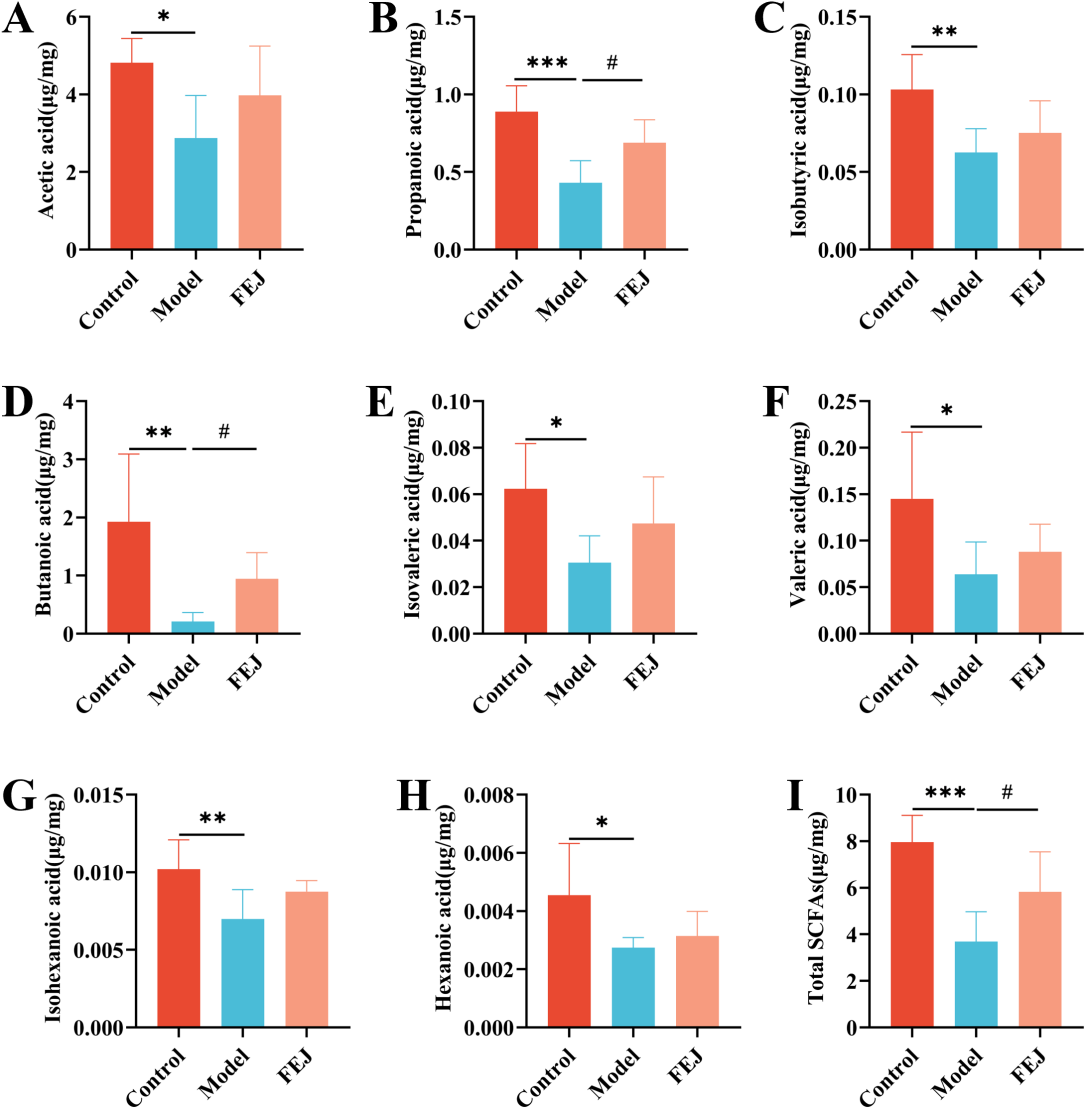
FEJ promoted the production of SCFAs in feces contents. **(A)** Acetic acid. **(B)** Propionic acid. **(C)** Isobutyric acid. **(D)** Butanoic acid. **(E)** Isovaleric acid. **(F)** Valeric acid. **(G)** Isohexanoic acid. **(H)**Hexanoic acid and **(I)** Total SCFAs. Values were expressed as means ± SD (n=6). ^*^ *p*< 0.05, ^**^ *p* < 0.01 and ^***^ *p*< 0.001 versus control group. ^#^ *p* < 0.05 versus model group.

Acetic acid, propionic acid, isobutyric acid, butanoic acid, isovaleric acid, valeric acid,isohexanoic acid, hexanoic acid and total SCFAs levels were significantly decreased in the model groups compared to the control group (*p* < 0.05) (**Figures 3A–I**). Additionally, the FEJ group exhibited higher production of SCFAs compared to the model group. The most significantly abundant SCFAs were found to be propanoic acid, butanoic acids and total SCFAs(*p* < 0.05).

### Impact of FEJ on gut microbiome

**Figure 4:**
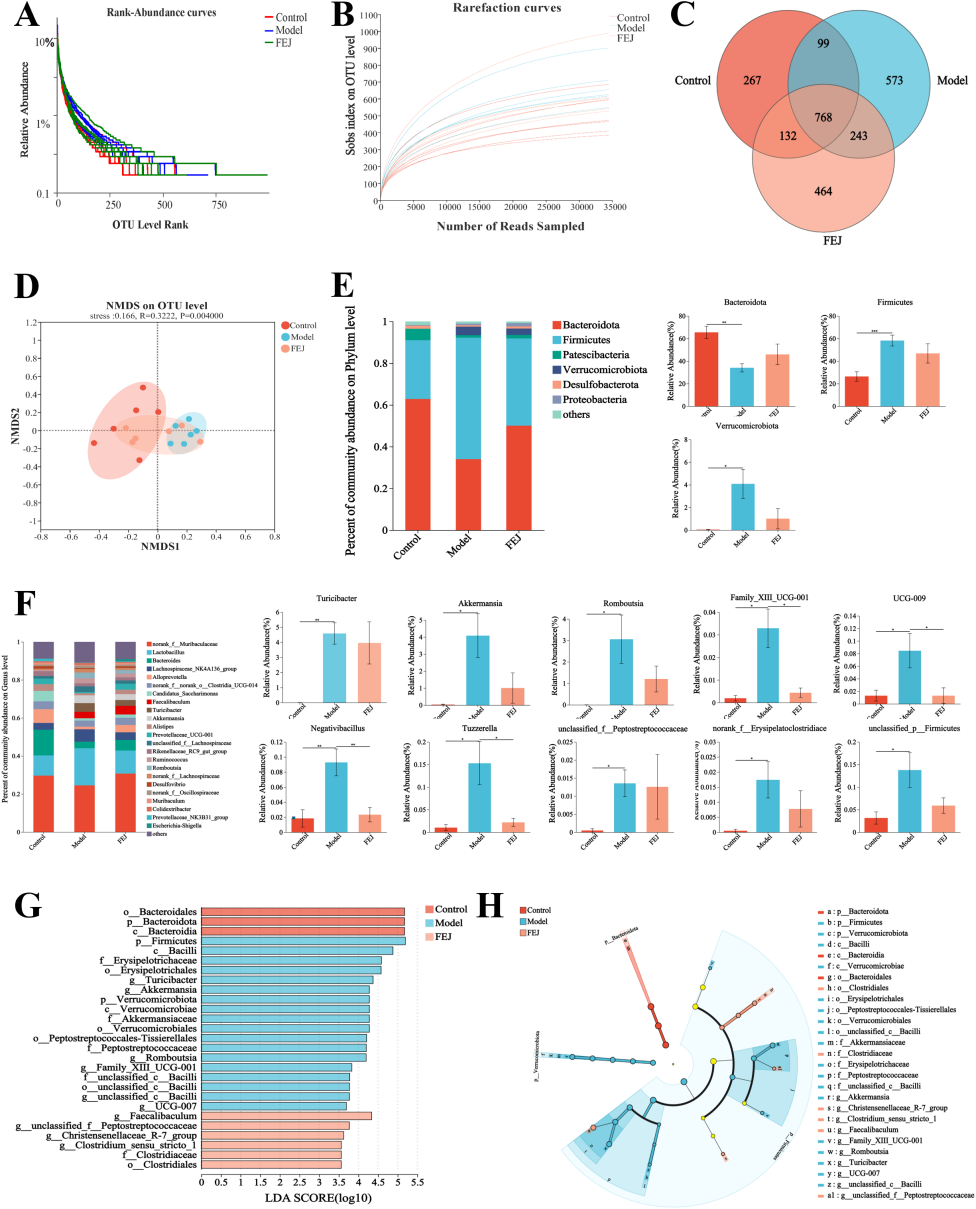
Impact of FEJ on gut microbiota (n= 6). **(A)** Rarefaction curves. **(B)** Rank abundance curves. **(C)** OTU numbers of Venn diagram **(D)** NMDS analysis at OTU level. **(E)** Relative abundance of gut microbiota composition at phylum level. **(F)** Relative abundance of gut microbiota composition at genus level. **(G)** LEfSe analysis with Cladogram and **(H)** LDA score (LDA > 3.5). *p< 0.05, **p < 0.01 and ***p< 0.001 indicated significant correlation.

The rank-abundance curves and the rarefaction curves (Sobs index on OTU level) exhibited a flat profile (**Figures 4A, B**), suggesting that the sequencing volume was reasonable and that all the types were successfully recovered. In the control, model, and FEJ groups, we identified a total of 1266, 1683 and 1607 OTUs, respectively, with 267, 573 and 464 unique OTUs identified in each group (**Figure 4C**). Principal coordinate analysis (PCoA) demonstrated that the gut bacterial community in the FEJ group (orange) closely resembled the control group (red). In contrast, the model group (blue) exhibited a more pronounced differentiation from the control group (**Figure 4D**).

**Figure 4E** illustrated a histogram of species abundance, highlighting that the predominant intestinal bacteria at the phylum level consisted mainly of *Bacteroidota*, *Firmicutes*, *Patescibacteria*, *Verrucomicrobiota*, *Proteobacteria* and *Desulfobacterota*, with *Bacteroidota* being the most dominant bacteria. Among them, the model group significantly down-regulated *Bacteroidota*, while up-regulated *Firmicutes* and *Verrucomicrobiota*. Conversely, FEJ treatment counteracted these changes, up-regulating *Bacteroidota* and down-regulating *Firmicutes* and *Verrucomicrobiota*.

As shown in **Figure 4F**, the distribution of genus level of intestinal bacteria was made up primarily of *norank_f_Muribaculaceae*, *Lactobacillus*, *Bacteroides*, *Lachnospiraceae_NK4A136_group*, *Alloprevotella*, *norank_f_norank_o_Clostridia_UCG-014*, *Candidatus_Saccharimonas*, *Faecalibaculum*, *Turicibacter* and *Akkermansia*. However, the abundance of *Turicibacter*, *Akkermansia*, *Romboutsia*, *Family_XIII_UCG-001*, *UCG-009*, *Negativibacillus*, *Tuzzerella*, *unclassified_f_Peptostreptococcaceae*, *norank_f_Erysipelatoclostridiaceae* and *unclassified_p_Firmicutes* significantly increased in the model group compared to the control group (*p* < 0.05). Following FEJ treatment, there was a notable decrease in the abundance of *Family_XIII_ UCG-001*, *UCG-009*, *Negativibacillus* and *Tuzzerella*(*p* < 0.05).

We used LEfSe analysis to determine the classified bacterial taxas that were altered. The LDA scores and taxonomic diagram are displayed in **Figures 4G, H**. Twenty-seven distinct classes of taxa were identified, exhibiting varying degrees of richnesses among three groups. *C_Bacteroidia* were the dominant microbiota in the control group, *p_Firmicutes* and *g_Turicibacter* were the dominant microbiota in the model group and *g_Faecalibaculum* was the primary microbiota in the FEJ group. Overall, these findings indicate that FEJ treatment reversed the composition and structure of the gut microbiota of CTX and APH-induced mice.

### Impact of FEJ on fecal metabolome

**Figure 5:**
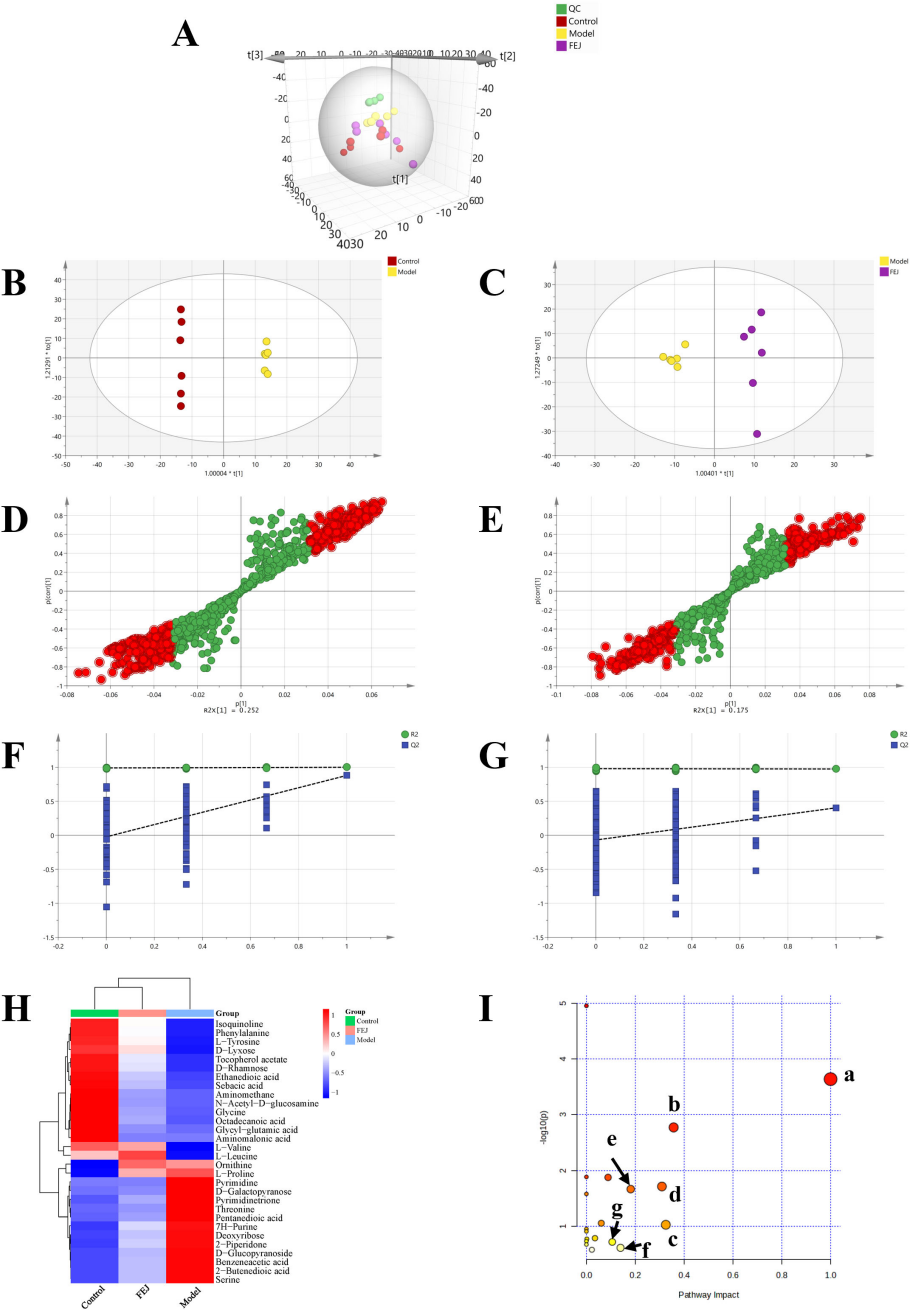
Impact of FEJ on fecal metabolic profile in anemia mice(n = 6). **(A)** The PCA score plots among all groups (R^2^X = 0.657, Q^2^ = 0.381). The OPLS-DA score plots between **(B)** the control and model groups (R^2^Y =1, Q^2^ = 0.879), **(C)** the model and FEJ groups (R^2^Y = 0.974, Q^2^ = 0.403). The permutation test analysis between **(D)** the control and model groups, **(E)** the model and FEJ groups. Score plot from OPLS-DA model classifying **(F)** the control and model groups, **(G)** the model and FEJ groups. **(H)** The heatmap of all the differential metabolites. **(I)** Summary of pathway analysis in the fecal. a-phenylalanine, tyrosine and tryptophan biosynthesis; b-phenylalanine metabolism; c-starch and sucrose metabolism; d-glycine, serine and threonine metabolism; e-arginine and proline metabolism; f-tyrosine metabolism; g-glyoxylate and dicarboxylate metabolism; KEGG, Kyoto Encyclopedia of Genes and Genomes.

Metabolites in fecal samples were analyzed from the control, model group and FEJ groups in order to overall assess the metabolomics regulation of FEJ intake in anemic mice. Principal component analysis (PCA) clearly indicated a trend of separation between the control group and the model group, suggesting differences in metabolites between the two groups. Additionally, the spatial distances of the FEJ group were closer to the control compared to the model group (**Figure 5A**). OPLS-DA based S-plot visualization is often used to explore different metabolites between groups and assess integrating VIP (>1), *p* (<0.05). The results demonstrated that the groups were well grouped as shown in **Figures 5B–E**. A permutation test was conducted on the model to prevent overfitting and the results indicated that the model was applicable (**Figures 5F, G**). Following a comparison with the database, a total of 30 and 16 differential metabolites were identified between the model and FEJ groups, respectively (**Table 1**). The variation of all differential metabolites in the two comparison groups (the control group compared with the model group and the model group compared with the FEJ group) was displayed in **Figure 5H**. The two comparison groups shared 15 differential metabolites. After FEJ treatment of anemic mice, five metabolites were upregulated, such as ethanedioic acid, D-glucopyranoside, octadecanoic acid, sebacic acid and isoquinoline, whereas ten metabolites were downregulated, such as L-valine, L-leucine, L-proline, glycine, threonine, 2-piperidone, ornithine, phenylalanine, pyrimidinetrione and L-tyrosine.

**Table 1:** Differentia metabolites among the control group, model group, and FEJ group.

NO	Metabolites	Formula	RT (min)	*m/z*	*p*(Con vs Mod)	VIP(Con vs Mod)	Trend(Con vs Mod)	Trend (Mod vs FEJ)
1	ethanedioic acid	C_2_H_2_O_4_	11.16	73.1	0.029	1.2300	↓^*^	↑^*^
2	L-valine	C_5_H_11_NO_2_	12.86	145.1	0.002	1.8429	↑^**^	↓^**^
3	L-leucine	C_6_H_13_NO_2_	13.98	159.1	0.002	1.6967	↑^**^	↓^*^
4	L-proline	C_5_H_9_NO_2_	14.50	142.0	0.011	1.4305	↑^*^	↓^*^
5	glycine	C_2_H_5_NO_2_	14.64	174.0	0.000	1.9300	↑^****^	↓^**^
6	threonine	C_4_H_9_NO_3_	15.92	117.0	0.002	1.5396	↑^**^	↓^*^
7	2-piperidone	C_5_H_9_NO	17.02	128.0	0.000	1.4375	↑^****^	↓^*^
8	ornithine	C_5_H_12_N_2_O_2_	19.09	142.0	0.004	1.5736	↑^**^	↓^*^
9	phenylalanine	C_9_H_11_NO_2_	19.28	219.0	0.000	1.9824	↑^****^	↓^**^
10	pyrimidinetrione	C_4_H_4_N_2_O_3_	21.46	256.1	0.005	1.8164	↑^**^	↓^*^
11	D-glucopyranoside	C_6_H_12_O_6_	21.70	204.0	0.014	1.1096	↓^**^	↑^*^
12	L-tyrosine	C_9_H_11_NO_3_	22.68	219.0	0.003	1.5188	↑^**^	↓^*^
13	octadecanoic acid	C_18_H_36_O_2_	25.91	73.1	0.000	1.8535	↓^****^	↑^*^
14	sebacic acid	C_10_H_18_O_4_	28.05	331.1	0.022	1.5156	↓^*^	↑^*^
15	isoquinoline	C_9_H_7_N	30.39	192.0	0.000	2.3461	↓^****^	↑^**^

^*^ *p* < 0.05, ^**^ *p* < 0.01 and ^****^ *p* < 0.0001 indicated significant correlation. VIP value was obtained from the OPLS-DA model.

The symbol ↑ (FC > 1) up or ↓ (FC < 1) down arrows represent the relatively increased or decreased levels of the metabolites, respectively.

Subsequently, the significant metabolites were conducted for metabolic pathway analysis via metaboanalyst. Seven primary altered pathways were identified in the model vs. control/FEJ groups (**Figure 5I**, impact value > 0.1) as follows: phenylalanine, tyrosine and tryptophan biosynthesis, glycine, serine and threonine metabolism, phenylalanine metabolism, starch and sucrose metabolism, arginine and proline metabolism, glyoxylate and dicarboxylate metabolism and tyrosine metabolism.

### Impact of FEJ on spleen transcriptome

**Figure 6:**
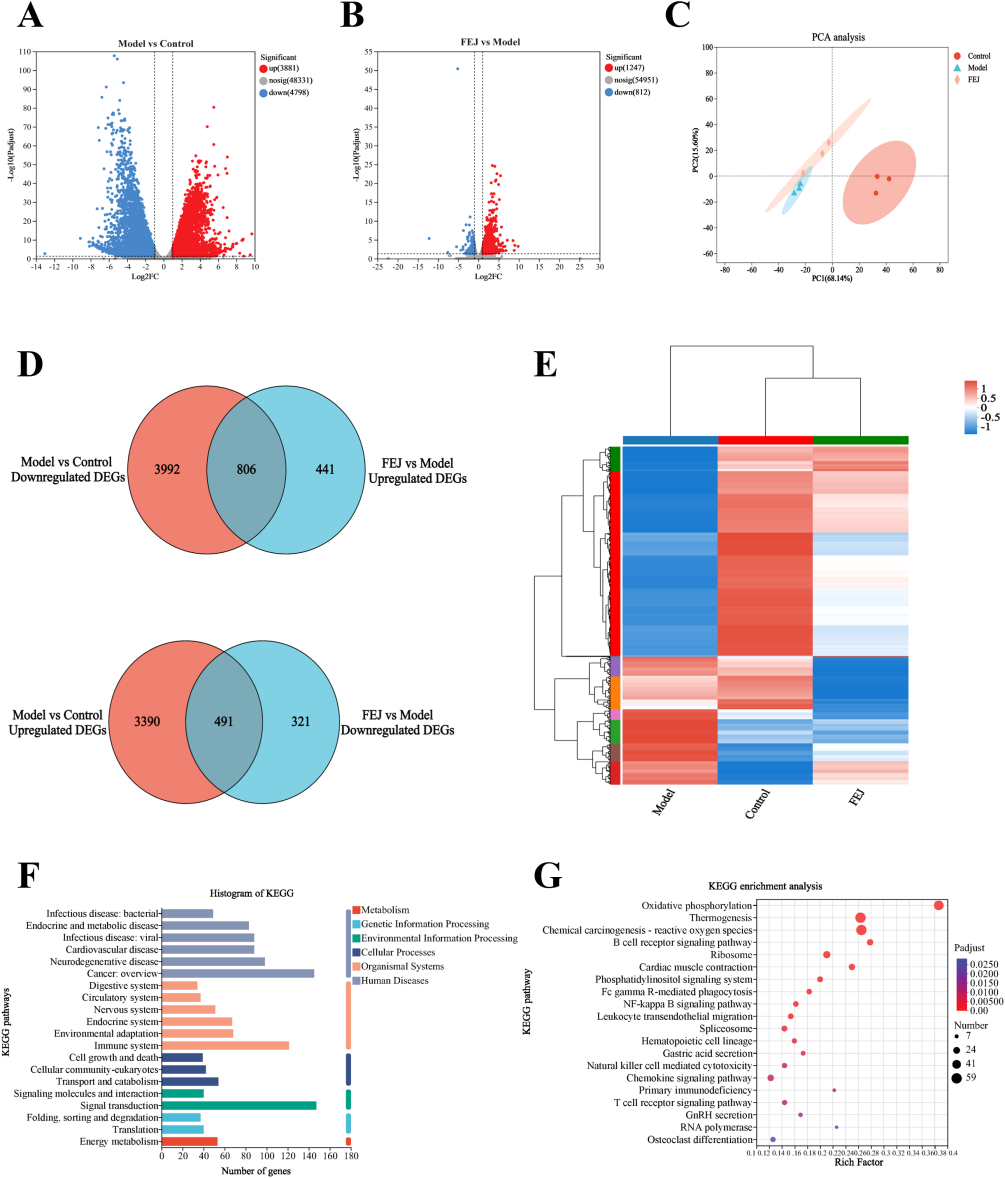
Impact of FEJ on spleen transcriptome profile in anemia mice. Volcano plots of differentially expressed genes in **(A)** the control group vs model group, **(B)** the model group vs FEJ group (DEGs, n = 3 mice per group). **(C)** PCA among the three groups. **(D)**Venn diagram of up-regulated and down-regulated DEGs among the three groups. **(E)** Heatmap showing the cluster analysis of DEGs. **(F)** Functional annotation of DEGs among the three groups. **(G)** KEGG pathway enrichment analysis of DEGs among the three groups. KEGG, Kyoto Encyclopedia of Genes and Genomes.

We performed transcriptome analysis on spleen tissues from the control, model and FEJ groups to interpret the effects of FEJ on anemia mice. This analysis revealed a total of 8679 DEGs between the model group and the control group, and 2059 DEGs in the FEJ group compared to the model group (**Figures 6A, B**). PCA revealed distinct clusters among the groups. The model group was distinguished from the control group, and the FEJ group showed intermediate variability between the control and model groups in **Figure 6C**. The Venn diagram and cluster analysis heat map revealed the restoration of 1297 DEGs after FEJ treatment, which were previously skewed by CTX/APH (**Figures 6D, E**). Based on the KEGG pathways classification, six, six, three, two, two and one pathways were associated with human diseases, organismal systems, cellular processes, environmental information processing, genetic information processing and metabolism pathways, respectively(**Figure 6F**). The KEGG enrichment analysis revealed that the DEGs reversing by FEJ were found significantly enriched in Fc gamma R-mediated phagocytosis, NF-κB signaling pathway, hematopoietic cell lineage, GnRH secretion, RNA polymerase, osteoclast differentiation and multiple immune-related signaling pathways (B cell receptor signaling pathway, leukocyte transendothelial migration, natural killer cell mediated cytotoxicity, chemokine signaling pathway, primary immunodeficiency and T cell receptor signaling pathway), etc (*p* < 0.05, **Figure 6G**).

### Analyses of the microbiome, metabolome and transcriptome correlations

#### Analysis of correlations between hematopoietic index and microbiome

**Figure 7:**
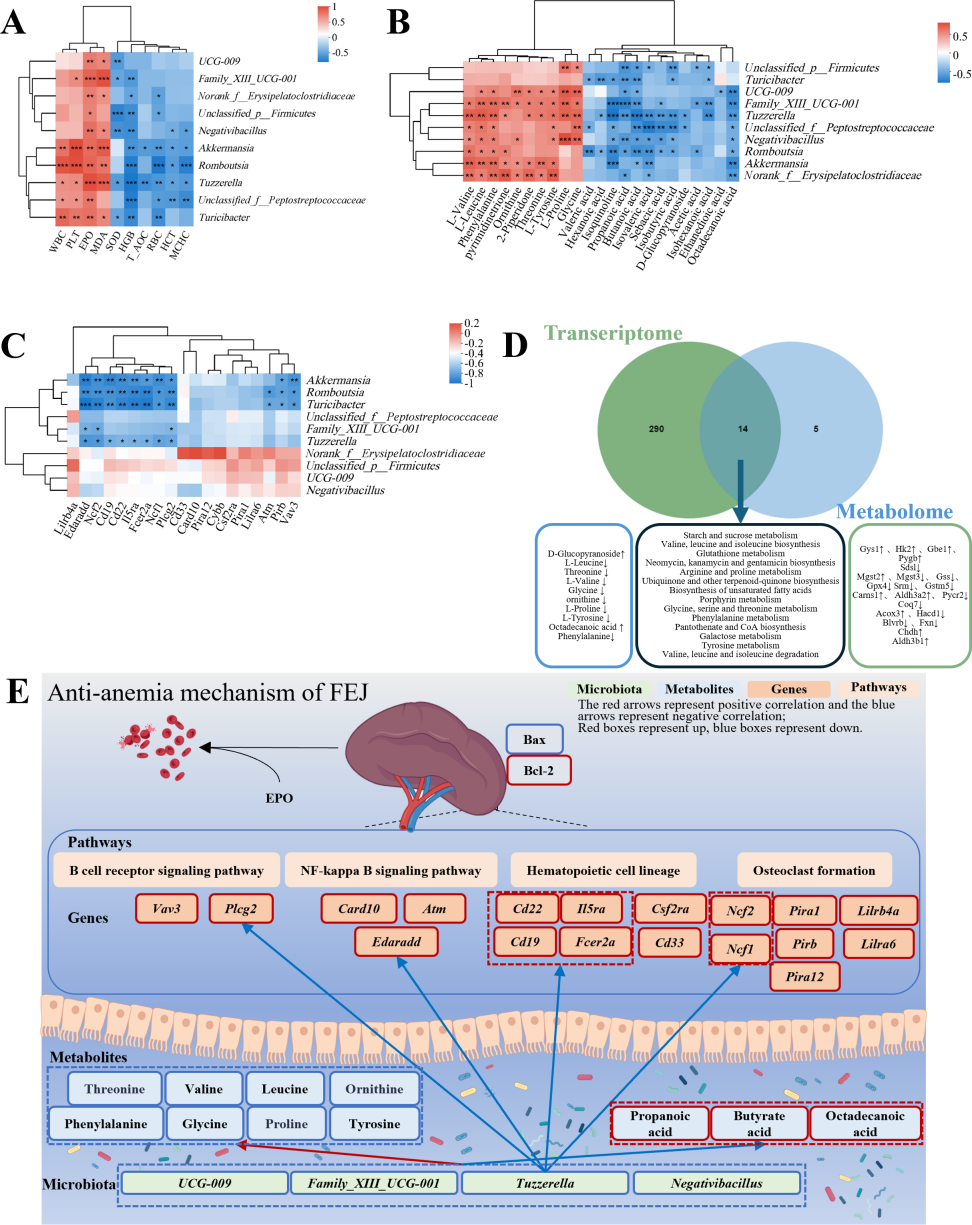
Associations amongst hematopoietic-related indicators, microbiome, metabolome and transcriptome influenced by FEJ. **(A)** Correlation heatmap between hematopoietic-related indicators and fecal bacteria at genus level. **(B)** Correlation heatmap between metabolites and fecal bacteria at genus level. **(C)** Correlation heatmap of genus-level fecal bacteria with top 20 DEGs in anemia-related pathways. **(D)** Combined metabolome and transcriptome analysis. **(E)** Schematic of FEJ resistance to the mechanism of CTX and APH-induced anemia. The color of each square represents negative correlation (blue) and positive correlation(red). Significance levels are indicated as ^*^ *p* < 0.05, ^**^ *p* < 0.01, and ^***^ *p* < 0.001.

Spearman’s correlation analysis was conducted to explore the connection between the hematopoietic-related traits and the genus level of the intestinal flora (**Figure 7A**). The richness of above mentioned ten kinds of different gut microbiota exhibited positive correlations with WBC, PLT, EPO and MDA while negative correlation with SOD, HGB, T-AOC, RBC, HCT and MCHC.

#### Analysis of correlations between microbiome and metabolome

FEJ influenced the gut microbiota composition and metabolic profiles, with Spearman’s correlation analysis revealing correlations between 15 significantly changed fecal metabolites and 10 gut microbiota at the genus level (**Figure 7B**). L-valine, L-leucine, phenylalanine, pyrimidinetrione, ornithine, 2-piperidone, threonine, L-tyrosine, L-proline and glycine were significantly positively correlated with 7, 8, 8, 5, 5, 5, 5, 7, 5 and 6 gut microbiota, respectively. Ethanedioic acid, octadecanoic acid, isoquinoline, D-glucopyranoside and sebacic acid were negatively correlated with 1, 8, 7, 2 and 5 gut microbiota.

Furthermore, the relationship between gut microbial composition and SCFAs production was also investigated. The concentrations of acetic acid, propanoic acid, isobutyric acid, butanoic acid, isovaleric acid, valeric acid, isohexanoic acid, hexanoic acid and total SCFAs were found to have a negative correlation with at least three representatives of fecal *Akkermansia*, *Unclassified_p_Firmicutes*, *Family_XIII_UCG-001*, *Romboutsia*, *Unclassified_f_Peptostreptococcaceae*, *Norank_f_Erysipelatoclostridiaceae*, *Tuzzerella*, *UCG-009*, *Negativibacillus* and *Turicibacter*.

#### Analysis of correlations between microbiome and transcriptome

According to *p*adjust value < 0.05, we screened out hematopoietic-related functional pathways, including B cell receptor signaling pathway, NF-κB signaling pathway, leukocyte transendothelial migration, hematopoietic cell lineage, T cell receptor signaling pathway and osteoclast differentiation. The top 20 DEGs were related to hematopoiesis pathways based on the Log2 FC (the FEJ group vs. the model group). Then, Spearman’s correlation analysis was conducted to examine the gut microbiota and the top 20 DEGs involved in hematopoietic pathways. As depicted in **Figure 7C**, Akkermansia, Romboutsia, Turicibacter, *Family_XIII_UCG-001* and *Tuzzerella* were notably associated with KEGG pathways implicated in anemia. For instance, *Romboutsia*, *Turicibacter* and *Tuzzerella* showed a significantly negative correlation with *Edaradd*, *Ncf2*, *Cd19*, *Cd22*, *I15ra*, *Fcer2a*, *Ncf1*, and *Plcg2*. Additionally, *Akkermansia*, *Romboutsia* and *Turicibacter* exhibited significantly negative correlations with *Pirb* and *Vav3*. Moreover, *Romboutsia* and *Turicibacter* were significantly negatively correlated with *Atm*, and *Family_XIII_UCG-001* was negatively correlated with *Edaradd*, *Ncf2*, and *Plcg2*.

#### Integrative analysis of metabolome and transcriptome

An integrative analysis of the transcriptome and metabolome was carried out to investigate the links between DEGs and metabolites. **Figure 7D** illustrated that 304 KEGG pathways were altered at the transcriptional level and 19 at the metabolome level. It was worth mentioning that 14 KEGG pathways were affected in both, among which nine pathways were associated with anemia, namely, starch and sucrose metabolism; valine, leucine and isoleucine biosynthesis; glutathione metabolism; arginine and proline metabolism; porphyrin metabolism; glycine, serine and threonine metabolism; phenylalanine metabolism; pantothenate and CoA biosynthesis; and tyrosine metabolism. Starch and sucrose metabolism belongs to carbohydrate metabolism. Valine, leucine and isoleucine biosynthesis, glutathione metabolism, arginine and proline metabolism, glycine, serine and threonine metabolism, phenylalanine metabolism and tyrosine metabolism belong to amino acid metabolism. Porphyrin metabolism and pantothenate and CoA biosynthesis belong to the metabolism of cofactors and vitamins.

## Discussion

The global burden of anemia is considerable, accounting for 8.8% of disability from all conditions (31). Anemia increases in prevalence with increasing age and frailty (32). Even without underlying health conditions, mild anemia is associated with impaired functional capacity, reduced physical performance and a diminished quality of life (33). People with anemia exhibit different traits from normal individuals, including body weight, spleen, liver, hematopoietic cytokines, fecal microbes, host genes and metabolites (34, 35). We observed that FEJ had beneficial effects on anemia induced by CTX and APH in mice and investigated its antianemia mechanism through microbiome, metabolome, and transcriptome analyses.

After modeling, the anemia mice exhibited a poor appearance, including easily shedding fluffy hairs, pale ears, noses, faces and feet, sleepiness, mobility retardation and emaciation. FEJ could markedly reverse the weight loss and appearance caused by anemia modeling. The blood routine data revealed that the peripheral RBC, HGB, HCT and MCHC of the model mice were decreased significantly, which are hallmark indicators of anemia. Meanwhile, WBC counts in the model group were higher than in the control group, indicating a compensatory leukocytosis triggered by CTX and APH (36). After treatment with FEJ, the peripheral blood cell count also showed a significant return to normal values. EPO is a hematopoietic growth factor which regulates blood cell production (37), mainly stimulating RBC production. EPO secretion generally helps to maintain normal levels of RBC and HGB for effective oxygen delivery to body (38). In regenerative anemia, elevated EPO levels respond to decreased HGB levels, reflecting a loss of erythrocytes due to hemolysis or bleeding (39). In our investigation, the EPO concentrations of serum samples in the model group were higher than in the control group, but decreased significantly with FEJ intervention, suggesting that FEJ may ameliorate hematopoietic damage by regulating EPO production. These findings implied that FEJ may facilitate hematopoietic rehabilitation in the model mice, which confirmed its traditional efficacy of tonifying blood.

It has been demonstrated that APH and CTX can impair the body’s antioxidant system, reducing its capacity for oxidative stress (40). T-AOC and SOD serve as pivotal indicators of antioxidant capacity. A dysregulation of oxidative stress response results in inadequate antioxidant capacity and lipid peroxidation, ultimately increasing the lipid peroxidation marker MDA, further aggravating bodily injury (41). FEJ increased the T-AOC and SOD levels in the liver while reducing MDA levels, indicating that FEJ enhanced antioxidant capacity in anemia mice.

The bone marrow has hematopoietic and immune defense functions, containing hematopoietic cells at various maturation stages (42). CTX can disrupt the blood-forming microenvironment, leading to inhibition of bone marrow hematopoiesis (15). FEJ could alleviate the severe damage of bone marrow in the histopathological lesions of the anemia model by decreasing adipocyte numbers and increasing hematopoietic cell counts in the bone marrow.

The thymus serves as a pivotal lymphoid organ responsible for the generation of T cells, which are essential for regulating both cellular and humoral immunity (43). The spleen is a critical organ for hematopoiesis, immunity and clearance of particulate matter from the blood, and its size and constituents are altered by anemia. The degrees of spleen enlargement are diagnostic features for anemia (44–47). Notably, FEJ successfully reversed the abnormalities in the size of the spleen and thymus. Moreover, FEJ was capable of restoring the spleen’s structural integrity to normal and facilitating the recovery of splenic function. Bax and Bcl-2 are apoptosis regulatory proteins. Bcl-2 can protect cells from mitochondrial damage and inhibit apoptosis, while Bax can increase mitochondrial membrane permeability, thereby promoting cell apoptosis (48). In the present investigation, apoptotic spleen cells and Bax expression were significantly increased and Bcl-2 expression was decreased in the model group. Treatment with FEJ notably normalized Bcl-2 and Bax expression, curtailing spleen cell apoptosis. Collectively, these observations imply that FEJ may promote the recovery of hematopoietic and immune function.

SCFAs, which are predominantly produced by the gut microbiome through the fermentation of complex carbohydrates, play a crucial role in maintaining intestinal homeostasis and barrier function (49). Acetic acid, propionic acid and butyric acids are the most abundant SCFAs. Anemic patients exhibit reduced SCFAs concentrations in feces or serum due to the impaired synthesis of SCFAs. Butyrate and supplementation with butyrate can restore iron metabolism to ameliorate anemia (50). Additionally, propionate can modulate the differentiation of bone marrow hematopoietic cells and effectively combat iron deficiency anemia (51). In this research, the concentrations of propanoic acid, butanoic acid and total SCFAs in fecal samples treated with FEJ were markedly elevated compared to those in the model group, suggesting that FEJ improved anemia by increasing the concentration of SCFAs.

Compared with the control group, some pathogenic bacteria exhibited significant changes in the model group, such as *Turicibacter*, *Akkermansia*, *Romboutsia*, *Desulfobacterota*, *Negativibacillus* and *Erysipelotrichaceae*. FEJ modulated the composition of the gut microbiota in anemia mice at the genus level, significantly decreasing the abundance of genera such as *Family_XIII_UCG-001*, *UCG-009*, *Negativibacillus* and *Tuzzerella*. *Turicibacter*, related to inflammation, has been linked to intestinal barrier disruption upon its elevation (52). *Akkermansia* was previously identified as a bacterium with probiotic properties and a health marker in healthy individuals. Multiple studies *in vivo* revealed its critical role in modulating inflammation, enhancing immune function, maintaining energy homeostasis, inhibiting pathogenic microbes, improving metabolic disorders, suppressing tumors, regulating autoimmune responses and alleviating psychological disorders. Clinical observations indicated a reduced abundance of *Akkermansia* in patients with diabetes, obesity, intestinal disorders and metabolic diseases compared to healthy individuals (53). However, other studies showed adverse effects associated with elevated abundance of *A*. *muciniphila*, including on the induction of Parkinson’s disease and ulcerative colitis (54). Additionally, the pathobionts *Akkermansia* expanded in the mice with severe anemia and monocytopenia (55), and overcolonization of *Akkermansia* was found to exacerbate intestinal diseases through disrupting the intestinal barrier by consuming excessive mucin (56).

The abundance of *A*. *muciniphila* significantly increased in 4-day fasted Syrian hamsters and 30-day fasted Burmese pythons, associated with dietary iron inadequate (57, 58). This mucin-degrading bacteria gained a competitive advantage during nutrient deprivation due to its ability to utilize mucin as a constant nutrient source. Host mucus production and secretion were correlated with dietary iron content. Furthermore, *A*. *muciniphila* exhibited advantage for growth in vehicle-treated mice with iron-deficiency anemia (IDA) induced by an iron-deficient diet. The study hypothesized that IV iron treatment elevated plasma iron levels, providing luminal iron source to other gut microbes and thereby reducing *A. muciniphila* abundance in iron-supplemented groups (59). These contradictory findings highlight the need for comprehensive investigations to validate the characteristics of *Akkermansia*. Additionally, *Romboutsia* proliferates in gastrointestinal disorders, including irritable bowel syndrome, and its levels is positively correlated with pro-inflammatory cytokines, such as TNF (60). The strain *Desulfovibrio desulfuricans* in the phylum *Desulfobacterota* can exacerbate intestinal permeability and boost systemic inflammation when administered orally (61, 62). Mice with obesity-related disorders have a high content of *Negativibacillus* in cecal (63). *Erysipelotrichaceae* strains are associated with colitogenic effects (64). The abundance of *Family_XIII_UCG-001* increases when inflammation occurs (65). The increased risk of disease is positively correlated with *UCG-009* abundance (66). *Tuzzerella* is associated with the development of IBD, as its upregulation can promote an inflammatory response and increase intestinal permeability (67). Consuming polystyrene microplastics can cause liver damage, leading to an increase in pathogenic *Tuzzerella* (67, 68). DHA-enriched phosphatidylcholine alleviate NAFLD by improving hepatic oxidative stress (MDA, SOD) and reducing the abundance of *Tuzzerella* (69).

In this study, Spearman’s correlation analysis was conducted to examine the relationship between gut microbiota and hematopoietic indicators. *Akkermansia* and *Tuzzerella* showed significant positive correlations with WBC, PLT, EPO and significant negative correlations with HGB, RBC, HCT and MCHC. In addition, a positive correlation was found between *Tuzzerella* and MDA, but a negative correlation was found between *Tuzzerella* and other oxidative stress indicators(T-AOC and SOD). The results suggested that *Akkermansia* and *Tuzzerella* may be potential markers for improving hematopoietic function. Therefore, FEJ may ameliorate anemia by inhibiting the proliferation of harmful bacteria and by modulating the structure of gut microbiota, thereby controlling inflammation, intestinal barrier and liver oxidative stress in mice.

Fecal metabolites include own metabolites of the host and the metabolites of gut microbiota, which can reveal the activity of microbial communities and their symbiotic relationship with the host. Fecal metabolomics based on GC-MS technology has been applied on the therapeutic effect of FEJ in CTX and APH-induced anemic mice for the first time in our study. The fecal metabolomics profile revealed a disorder in amino acid metabolism in the model group, predominantly affecting neutral and acidic amino acids with a polyanionic group structure. The presence of the negative ion group exerts a profound influence on the oxygen-binding capacity of Hb, facilitating its linkage with dextran (70). The elevated valine level in anemia rats reflects liver failure due to the usage of APH (70). Generally, valine and leucine collaborate to support normal body growth, assist in tissue repair, regulate blood sugar, and supply energy to the body (71). Moreover, the liver antioxidant activity is disrupted in anemia mice induced with CTX and APH, which could be related to the elevated glycine level (72). In addition, glycine plays a role in regulating serum iron and accelerating serum protein synthesis. Threonine has the function of enhancing immunity. It is reported that *Angelica sinensis* exerted blood enrichment by decreasing abnormally elevated glycine and threonine levels (73). FEJ could significantly decrease glycine and threonine, speculating that the FEJ nourished blood by regulating glycine, serine and threonine metabolism. Phenylalanine is an essential aromatic amino acid, and abnormally high levels of plasma phenylalanine in patients with severe aplastic anemia may cause bone marrow and hematopoietic stem cell injury (74). A damaged liver can’t catabolize phenylalanine and tyrosine, leading to elevated concentrations of them in individuals with liver disease (75). FEJ influenced the metabolic pathways of phenylalanine, tyrosine and tryptophan biosynthesis, in line with previous reports (76). By regulating the concentrations of these amino acids, FEJ potentially facilitated hemoglobin’s oxygen affinity, enhanced liver antioxidant activity, and improved the cellular microenvironment involved in anemia, suggesting hepatoprotective effects and hematopoietic support. Moreover, proline and ornithine were significantly different metabolites between the model group and the FEJ group, with their enrichment noted in the arginine and proline metabolism pathway. Arginine is capable of biosynthesizing both proline and ornithine (77, 78). Proline promotes the formation of osteoblastogenesis. It is speculated that FEJ regulated the formation and function of osteoblasts by participating in arginine and proline metabolism pathway. Methyl α-D-glucopyranoside, which has demonstrated a strong binding capacity to hemoglobin E in molecular docking studies, is a potential drug candidate for deficient hemoglobin synthesis in β-thalassemia (β-TH) (79). Octadecanoic acid, a beneficial saturated fatty acid, helps to maintain membrane dynamics. Low levels of octadecanoic acid can weaken the RBC membrane. Starch and sucrose metabolism along with dicarboxylate metabolism altered in β-TH patients (80), which is consistent with our findings.

In the correlation analysis, *UCG-009*, *Family_XIII_UCG-001*, *Tuzzerella* and *Negativibacillus* was found to be positively correlated with the levels of L-leucine, phenylalanine, L-proline and glycine and negatively correlated with the levels of octadecanoic acid. Anemia and blood deficiency are linked to a spectrum of metabolic disturbances including amino acid metabolism (16, 81, 82). These results suggested that FEJ may regulate phenylalanine metabolism, glycine, serine, and threonine metabolism, arginine and proline metabolism, as well as octadecanoic acid in anemic mice by influencing these gut microbiota. *Turicibacter*, *Tuzzerella*, *Negativibacillus* and *Romboutsia* were found to be significantly negatively correlated with propanoic acid and butanoic acid. Gut dysbiosis inhibited the production of SCFAs in thalassemia mice (83). The increase in the harmful bacteria *Turicibacter*, *Tuzzerella*, *Negativibacillus* and *Romboutsia* disrupted a balance of gut flora, resulting in decreased SCFAs content (84–88). SCFAs and anemia-related metabolic pathways interact closely with intestinal flora (89). The relative abundance of *Tuzzerella* and *Negativibacillus* were upregulated in anemia mice and FEJ treatment could significantly restore their abundance to normal levels. FEJ may alleviate anemia by down-regulating *Tuzzerella* and *Negativibacillus*, thereby promoting the production of SCFAs. The changes in gut microbiota induced by FEJ were likely to cause corresponding variations in metabolism, which emphasized the role of FEJ in modulating intestinal metabolism and maintaining gut microbiota homeostasis in anemic mice. Especially, targeted metabolomics of SCFAs based on GC-MS and 16S rRNA sequencing combining with Spearman’s correlation analysis of hematopoietic indices were firstly empolied to find potential targets.

A previous study showed that the nourishing blood mechanisms of E’jiao in bone marrow cells, as revealed via RNA sequencing in a 5-fluorouracil-induced myelosuppression mouse model, were associated with hematopoietic cell lineage, osteoclast differentiation, T cell receptor signaling pathways and NF-κB signaling pathways, etc (90). Spleen is one of the primary organs and is most susceptible to anemia. Spleen dysfunction is closely linked to blood deficiency and immune dysregulation. The accumulated bone marrow progenitor cells and RBC can induce splenic enlargement, exacerbating anemic symptoms (91). However, no RNA-seq-based study has been used in the therapeutic effect of FEJ on spleen function in CTX and APH-induced anemic mice. Among the in spleen pathways modulated by FEJ treatment, the B cell receptor signaling pathway stood out, involving *Plcg2* and *Vav3*. The knockdown *Plcg2* in megakaryocytes(Mks) reduces thrombopoietin (Thpo) expression, leading to decreased bone marrow hematopoietic stem cells (HSCs) quiescence and repopulation potential, as well as extramedullary hematopoiesis (92). *Vav3* is critical for B and T cells development and osteoclast function (93). FEJ also regulated the NF-κB signaling pathway, including *Edaradd*, *Card10* and *Atm. Edaradd* knockdown in tongue squamous cell carcinoma (TSCC) cells reduces proliferation and induces apoptosis (94), while *Atm* deficiency disrupts hematopoiesis and reduces myeloid and lymphoid hematopoietic cells in zebrafish (95, 96). Transcriptomic data showed FEJ significantly modulated hematopoietic cell lineage biomarkers (*Cd19*, *Cd22*, *Il5ra*, *Fcer2a*, *Cd33* and *Csf2ra*), which are pivotal for hematopoietic cells maturation and specialization (97, 98). *Cd19* promotes B lymphocyte maturation and activation (99), while *Cd22* negatively regulates B-cell signaling pathways (100, 101). *Il5ra and Fcer2a* promote eosinophil development and neutrophil apoptosis, respectively (102, 103). FEJ also regulated the osteoclast formation including *Lilrb4a*, *Ncf2*, *Ncf1*, *Pira12*, *Pira1*, *Lilra6* and *Pirb*. Endochondral ossification is tightly associated with the generation of the hematopoietic stem cell in bone marrow (104). *Ncf2* indirectly promotes osteoclast differentiation (105), and mutations in *Ncf2* or *Ncf1* are correlated with chronic granulomatosis (CGD), a genetic disorder often accompanied by anemia (106). *Pirb* supports the stemness of hematopoietic stem and progenitor cells (HSPCs) in hematopoietic organs (107). The FEJ group restored EPO levels and *OsM* expression, potentially through modulation of hematopoietic cell lines and osteoclastic bone marrow. Elevated EPO levels represent the physiological response to anemia (108). Animals lacking the Oncostatin M (OsM) receptor suffer from anemia (109, 110) and EPO stimulation activates OsM in erythroblasts, thereby stimulating osteoclast differentiation (108, 111, 112).

The Spearman’s correlation analysis revealed significant negative correlations between intestinal flora including *Akkermansia*, *Romboutsia*, *Turicibacter*, *Family_XIII_UCG-001* and *Tuzzerella*, and biomarker genes in B cell receptor signaling pathway, NF-κB signaling pathway, hematopoietic cell lineage and osteoclast differentiation. Benzene exposure deregulates the B cell receptor signaling pathway by down-regulating *CD22*, inhibiting NF-κB activation, causing an oxidative stress imbalance, thereby ultimately inhibiting spleen cell proliferation and promoting spleen cell apoptosis (113). It was reported that decreased *Tuzzerella* abundance is associated with relieved hepatic oxidative stress (MDA, SOD) (69), consistent with our findings. We identified that *Tuzzerella* has a significant correlation in regulating hematopoietic function and oxidative stress using Spearman’s correlation analysis. Therefore, *Tuzzerella* may regulate oxidative stress and splenic injury to reduce anemia through various signaling pathways.

The correlation analysis between transcriptome and metabolome profiles indicated that twenty-one DEGs and ten differential metabolites were enriched in fourteen anemia-related pathways. Microsomal glutathione S-transferase 2(*Mgst2*), glutathione synthetase (*Gss*), glutathione peroxidase 4 (*Gpx4*) and glutathione S-transferase (*Gst*), mu 5 (*Gstm5*) are differential genes in glutathione metabolism. *Mgst2* can mitigate ROS-mediated lipid peroxidation. The direct binding of coniferyl ferulate to *Mgst2* alleviates the toxic effect of xylene on HSPCs induced by oxidative stress (114). Variants in *Gss* can reduce *Gss* activity, leading to lower *GSH* levels, which in turn cause red blood cell rupture and anemia (115). Mice exhibit anemia and ineffective erythropoiesis when *Gpx4* is deletion (116). *Gstm5* is associated with refractory anemia with excess blast progression (117). Starch and sucrose metabolism requires the participation of glycogen synthase 1 (*Gys1*) and hexokinase2(*Hk2*). Enarodustat protects against renal anemia by increasing the expression of *Gys1*, leading to upregulated glycogen synthesis (118). *Angelica sinensis* polysaccharides facilitate the expression of the glycolytic gene *Hk2*, which promoted splenic glycolysis and extramedullary stress erythropoiesis, thereby ameliorating anemia (119). Choline dehydrogenase(*Chdh*) is a differential gene in glycine, serine and threonine metabolism. In the hemopoietic systems, *Chdh* exhibits significant enzyme activity in granulopoietic cells, while erythroblasts, megakaryocytes and platelets show only weak positive activity. These results are related to cellular lipid metabolism and blood cell functions (120). Integrative analysis of transcriptomic and metabolomic data indicated that FEJ prevented anemia by interfering with amino acid metabolism and carbohydrate metabolism.

## Conclusion

This study comprehensively investigated the hematopoietic characteristics of FEJ for the first time by integrating multi-omics analysis of fecal metabolomics, intestinal microbiota and spleen transcriptome profiles in CTX and APH-induced anemic mice. FEJ exhibited beneficial effects on hematological parameters, organ indexes, peripheral blood routine, hepatic oxidative stress and tissue damage in the bone and spleen. FEJ effectively ameliorated gut microbiome dysbiosis by reducing the abundance of pathogenic genera, including *Family_XIII_UCG-001*, *UCG-009*, *Negativibacillus* and *Tuzzerella* in anemia mice, while establishing *Akkermansia* and *Tuzzerella* as potential microbial markers for hematopoietic enhancement. Distinct metabolic profile variations revealed key fecal biomarkers including propanoic acid, butanoic acid, L-leucine, phenylalanine, L-proline, glycine and octadecanoic acid, which potentially regulated phenylalanine metabolism, glycine, serine and threonine metabolism, arginine and proline metabolism. Transcriptome analysis identified spleen hematopoietic regulators, including B cell receptor signaling pathway (*Plcg2*, *Vav3*), NF-κB signaling pathway (*Edaradd*), hematopoietic cell lineage biomarkers (*Cd19*, *Cd22*, *Il5ra*, *Fcer2a*) and osteoclast formation (*Ncf2*, *Ncf1*, *Lilra6*, *Pirb*), as potential diagnostic indicators (**Figure 7E**). The Spearman’s correlation analysis revealed significant associations between hematopoietic indicators and the gut microbiota, with functional connections between microbial communities and hematopoietic related genes and amino acid metabolism. An integrated metabolome and transcriptome analysis demonstrated FEJ’s regulatory impact on metabolic disturbances induced by anemia, particularly amino acid metabolism and carbohydrate metabolism. The results indicated FEJ ameliorated anemia through regulating the amino acid metabolism, inhibiting the proliferation of harmful bacteria, controlling inflammation and liver oxidative stress and reflecting the multi-target therapeutic effect of traditional Chinese medicine. These findings offer valuable insights into application of FEJ as a healthy food and Chinese patent medicines for anemia treatment.

## Data Availability

The data presented in the study are deposited in the NCBI repository (https://www.ncbi.nlm.nih.gov/), accession number PRJNA1203393 and PRJNA1203422.

## References

[1] Wan D Liang XJ Yang LM He D Du Q Zhang WP. Integration of gut microbiota and metabolomics for the hematopoiesis of siwu paste on anemia rats. Heliyon. (2023) 9:e18024. doi: 10.2139/ssrn.4356666 37449126 PMC10336798

[2] Liu MN Ye XW Zhang SY Cheng SQ Wang X Li CS. Homotherapy for heteropathy of alzheimer’s disease and anemia through reducing the expression of toll-like receptor and TNF by steamed panax notoginseng. Biomed Pharmacother. (2023) 165:115075. doi:10.1016/j.biopha.2023.115075 37385213

[3] Chen ZW Chen XY Guo LP Cui XM Qu Y Yang XY. Effect of different cooking methods on saponin content and hematopoietic effects of panax notoginseng-steamed chicken on mice. J Ethnopharmacol. (2023) 311:116434. doi: 10.1016/j.jep.2023.116434 37030555

[4] Fei CH Xue QQ Li WJ Xu Y Mou LY Li WD. Variations in volatile flavor compounds in crataegi fructus roasting revealed by E-nose and HS-GC-MS. Front Nutr. (2023) 9:1035623. doi: 10.3389/fnut.2022.1035623 36761989 PMC9905410

[5] Chao CH Hsu JL Chen MF Shih YH Lee CH Wu ML. Anti-hypertensive effects of radix rehmanniae and its active ingredients, *Nat* . Prod Res. (2020) 34:1547–52. doi: 10.1080/14786419.2018.1516660 30580622

[6] Li W Wang YP Han JB Zhang J Li B Qi XD. UPLC-Q-TOF-MS and UPLC-QQQ-MS were used for the qualitative and quantitative analysis of oligosaccharides in fufang ejiao syrup. J Pharm Biomed Anal. (2023) 224:115193. doi: 10.1016/j.jpba.2022.115193 36521307

[7] Xue QL Miao PQ Miao KH Yu Y Li Z. An online automatic sorting system for defective ginseng radix et rhizoma rubra using deep learning. Chin Herb Med. (2023) 15:447–56. doi: 10.1016/j.chmed.2023.01.001 PMC10394327 37538869

[8] Liu MX Tan HN Zhang XK Liu Z Cheng YN Wang DL. Hematopoietic effects and mechanisms of Fufang E'jiao Jiang on radiotherapy and chemotherapy-induced myelosuppressed mice. J Ethnopharmacol. (2014) 152:575–84. doi: 10.1016/j.jep.2014.02.012 24534527

[9] Shi Q Chen ZJ Yang J Liu XX Su YJ. Review of codonopsis radix biological activities: a plant of traditional chinese tonic, *J* . Ethnopharmacol. (2024) 332:118334. doi: 10.1016/j.jep.2024.118334 38740108

[10] Shen L Chen H Zhu Q Wang Y Wang S Qian J. Identification of bioactive ingredients with immuno-enhancement and anti-oxidative effects from fufang-ejiao-syrup by LC–MS*n* combined with bioassays. J Pharm Biomed Anal. (2016) 117:363–71. doi: 10.1016/j.jpba.2015.09.024 26433168

[11] Zhang Y Ye TT Hong ZO Gong SQ Zhou XS Liu HB. Pharmacological and transcriptome profiling analyses of fufang E’jiao jiang during chemotherapy-induced myelosuppression in mice. J Ethnopharmacol. (2019) 238:111869. doi: 10.1016/j.jep.2019.111869 30978457

[12] Xiong Y Chen LJ Man JH Hu YP Cui XM. Chemical and bioactive comparison of panax notoginseng root and rhizome in raw and steamed forms. J Ginseng Res. (2019) 43:385–93. doi: 10.1016/j.jgr.2017.11.004 PMC6606817 31308810

[13] Gao M Zhang ZJ Zhang YM Li MH Che XY Cui XM. Steamed *panax notoginseng* attenuates renal anemia in an adenine-induced mouse model of chronic kidney disease. J Ethnopharmacol. (2022) 288:114941. doi: 10.1016/j.jep.2021.114941 35007683

[14] Duan LH Li QH Lu D Wang Y Cui C. Optimization of processing technology and investigation of hematopoiesis activity of red Notoginseng Radix et Rhizoma. Chin Tradit Pat Med. (2024) 46:48–54. doi:10.3969/j.issn.1001-1528.2024.01.009

[15] Du Q He D Zeng HL Liu J Yang H Xu LB. Siwu paste protects bone marrow hematopoietic function in rats with blood deficiency syndrome by regulating TLR4/NF-κB/NLRP3 signaling pathway. J Ethnopharmacol. (2020) 262:113160. doi: 10.1016/j.jep.2020.113160 32736053

[16] He D Dan W Du Q Shen BB Chen L Fang LZ. Integrated network pharmacology and metabolomics analysis to reveal the potential mechanism of siwu paste on aplastic anemia induced by chemotherapy drugs. Drug Des Dev Ther. (2022) 16:1231–54. doi: 10.2147/DDDT.S327433 PMC9061215 35517983

[17] Ji P Wei Y Hua Y Zhang X Yao W Ma Q. A novel approach using metabolomics coupled with hematological and biochemical parameters to explain the enriching-blood effect and mechanism of unprocessed angelica sinensis and its 4 kinds of processed products. J Ethnopharmacology. (2018) 211:101–16. doi: 10.1016/j.jep.2017.09.028 28958590

[18] Wang YY Zhou N Shan ZF Ke YY Liu Z Liu ZH. Metabolomic strategies and biochemical analysis of the effect of processed rehmanniae radix extract on a blood-deficient rat model. BMC Complementary Med Ther. (2022) 22:89. doi: 10.1186/s12906-022-03560-x PMC8957163 35337319

[19] Xu YL Zeng FQ Jiang JP Huo J Zhao CJ Yan ZG. The hematopoietic function of medicinal wine maoji jiu revealed in blood deficiency model rats. Evidence-Based Complementary Altern Med. (2022) 2022:1025504. doi: 10.1155/2022/1025504 PMC9325634 35911170

[20] Zhao XH Zhang YT Song X Yin ZQ Jia RY Zhao XF. Effect of chuanminshen violaceum polysaccharides and its sulfated derivatives on immunosuppression induced by cyclophosphamide in mice. Int J Clin Exp Med. (2015) 8:558–68. PMC4358485 25785030

[21] Zou YT Zhou J Zhu JH Wu C-Y Shen H Zhang W. Gut microbiota mediates the protective effects of traditional chinese medicine formula qiong-yu-gao against cisplatin-induced acute kidney injury. Microbiol Spectr. (2022) 10:e00759-22. doi: 10.1128/spectrum.00759-22 35481834 PMC9241845

[22] Liu CS Zhao DF Ma WJ Guo YD Wang AJ Wang QL. Denitrifying sulfide removal process on high-salinity wastewaters in the presence of halomonas sp. Appl Microbiol Biotechnol. (2016) 100:1421–6. doi: 10.1007/s00253-015-7039-6 26454867

[23] Edgar RC. UPARSE: highly accurate OTU sequences from microbial amplicon reads, *Nat* . Methods. (2013) 10:996–8. doi:10.1038/nmeth.2604 23955772

[24] Li Y Zong JX Ye WJ Fu YF Gu XY Pan WS. Pithecellobium clypearia: amelioration effect on imiquimod-induced psoriasis in mice based on a tissue metabonomic analysis, *Front* . Pharmacol. (2021) 12:748772. doi: 10.3389/fphar.2021.748772 PMC8484644 34603060

[25] Chen SF Zhou Y Chen YR Gu J. fastp: an ultra-fast all-in-one FASTQ preprocessor. Bioinf. (2018) 34:i884–90. doi: 10.1093/bioinformatics/bty560 PMC6129281 30423086

[26] Kim D Langmead B Salzberg SL. HISAT: a fast spliced aligner with low memory requirements. Nat Methods. (2015) 12:357–60. doi: 10.1038/nmeth.3317 PMC4655817 25751142

[27] Pertea M Pertea GM Antonescu CM Chang TC Mendell JT Salzberg SL. StringTie enables improved reconstruction of a transcriptome from RNA-seq reads. Nat Biotechnol. (2015) 33:290–5. doi: 10.1038/nbt.3122 PMC4643835 25690850

[28] Li B Dewey CN. RSEM: accurate transcript quantification from RNA-seq data with or without a reference genome. BMC Bioinf. (2011) 12:323. doi: 10.1186/1471-2105-12-323 PMC3163565 21816040

[29] Love MI Huber W Anders S. Moderated estimation of fold change and dispersion for RNA-seq data with DESeq2. Genome Biol. (2014) 15:550. doi: 10.1186/s13059-014-0550-8 25516281 PMC4302049

[30] Li Y Teng M Yang HX Li SY Liu X Zhang JC. Impact of macrophage differentiation on hematopoietic function enhancement by shenzhu ErKang syrup. Aging. (2024) 15:169–90. doi: 10.18632/aging.205358 PMC10817372 38175693

[31] Kassebaum NJ Jasrasaria R Naghavi M Wulf SK Johns N Lozano R. A systematic analysis of global anemia burden from 1990 to 2010. Blood. (2014) 123:615–24. doi: 10.1182/blood-2013-06-508325 PMC3907750 24297872

[32] Clevenger B Richards T. Pre-operative anemia. Anesthesia. (2015) 70:20–e8. doi: 10.1111/anae.2014.70.issue-s1 25440391

[33] Geisel T Martin J Schulze B Schaefer R Bach M Virgin G. An etiologic profile of anemia in 405 geriatric patients. Anemia. (2014) 2014:932486. doi: 10.1155/2014/932486 24707396 PMC3953485

[34] Chen HX Wu WG Tang SM Fu R Gong X Hou H. Altered fecal microbial and metabolic profile reveals potential mechanisms underlying iron deficiency anemia in pregnant women in China, 6. Biomol Biomed. (2022) 22:923–33. doi: 10.17305/bjbms.2022.7091 PMC9589311 35803345

[35] Galanello R Origa R. Beta-thalassemia. Orphanet J Rare Dis. (2010) 5:11. doi: 10.1186/1750-1172-5-11 20492708 PMC2893117

[36] Wang J Wang FR Yuan LX Ruan HS Zhu ZB Fan XL. Blood-enriching effects and immune-regulation mechanism of steam-processed polygonatum sibiricum polysaccharide in blood deficiency syndrome mice. Front Immunol. (2022) 13:813676. doi: 10.3389/fimmu.2022.813676 35250989 PMC8892585

[37] Bociek RG Armitage JO. Hematopoietic growth factors. Ca-Cancer J Clin. (1996) 46:165–84. doi: 10.3322/canjclin.46.3.165 8646546

[38] Turner J Parsi M Badireddy M. Anemia. StatPearls. (2024) NBK499994. 29763170

[39] Cotoraci C Ciceu A Sasu A Hermenean A. Natural antioxidants in anemia treatment, 4. Int J Mol Sci. (2021) 22:1883. doi: 10.3390/ijms22041883 33668657 PMC7918704

[40] Zeng FQ Xu YL Li YL Yan ZG Li L. Metabonomics study of the hematopoietic effect of medicinal wine maoji jiu on a blood deficiency rat model by ultra-high-performance liquid chromatography coupled to quadrupole time-of-flight mass spectrometry and a pattern recognition approach. Mol. (2022) 27:3791. doi: 10.3390/molecules27123791 PMC9227738 35744917

[41] Jiang SQ Zhang ZW Huang FF Yang ZS Yu FM Tang YP. Protective effect of low molecular weight peptides from solenocera crassicornis head against cyclophosphamide-induced nephrotoxicity in mice via the Keap1/Nrf2 pathway. Antioxidants. (2020) 9:745. doi: 10.3390/antiox9080745 32823691 PMC7465301

[42] Dou L Gong X Wu Q Mou FZ. Therapeutic effects of sheng xue fang in a cyclophosphamide-induced anemia mouse model. Pharm Biol. (2021) 59:789–98. doi: 10.1080/13880209.2021.1941133 PMC8238071 34176428

[43] Al-Suhaimi EA Aljafary MA Alkhulaifi FM Aldossary HA Alshammari T AL-Qaaneh A. Thymus gland: a double edge sword for coronaviruses. Vaccines. (2021) 9:1119. doi: 10.3390/vaccines9101119 34696231 PMC8539924

[44] Kho S Siregar NC Qotrunnada L Fricot A Sissoko A Shanti PAI. Retention of uninfected red blood cells causing congestive splenomegaly is the major mechanism of anemia in malaria. Am J Hematol. (2024) 99:223–35. doi: 10.1002/ajh.27152 PMC10952982 38009287

[45] Nardo-Marino A Glenthøj A Brewin JN Petersen J Braunstein TH Kurtzhals JAL. The significance of spleen size in children with sickle cell anemia. Am J Hematol. (2022) 97:1520–8. doi: 10.1002/ajh.v97.12 PMC9827862 36054667

[46] Shi XQ Zhu ZH Yue SJ Tang YP Chen YY Pu ZJ. Integration of organ metabolomics and proteomics in exploring the blood enriching mechanism of danggui buxue decoction in hemorrhagic anemia rats. J Ethnopharmacol. (2020) 261:113000. doi: 10.1016/j.jep.2020.113000 32663590

[47] Al-Salem AH. Splenic complications of sickle cell anemia and the role of splenectomy. ISRN Hematol. (2011) 2011:864257. doi: 10.5402/2011/864257 22084706 PMC3200071

[48] Lalier L Vallette F Manon S. Bcl-2 family members and the mitochondrial import machineries: the roads to death. Biomolecules. (2022) 12:162. doi: 10.3390/biom12020162 35204663 PMC8961529

[49] Ma TT Li C Zhao FQ Cao J Zhang XY Shen XR. Effects of co-fermented collagen peptide-jackfruit juice on the immune response and gut microbiota in immunosuppressed mice. Food Chem. (2021) 365:130487. doi: 10.1016/j.foodchem.2021.130487 34237564

[50] Xiao P Cai XC Zhang Z Guo K Ke Y Hu ZW. Butyrate prevents the pathogenic anemia-inflammation circuit by facilitating macrophage iron export. Adv Sci (Weinh). (2024) 11:2306571. doi: 10.1002/advs.202306571 38235606 PMC10966513

[51] Li Y Han M Song J Liu SJ Wang YJ Su XH. The prebiotic effects of soluble dietary fiber mixture on renal anemia and the gut microbiota in end-stage renal disease patients on maintenance hemodialysis: a prospective, randomized, placebo-controlled study. J Transl Med. (2022) 20:599. doi: 10.1186/s12967-022-03812-x 36517799 PMC9753397

[52] Rahman AT Shin J Whang CH Jung W Yoo D Seo CJ. Bilirubin nanomedicine rescues intestinal barrier destruction and restores mucosal immunity in colitis. ACS Nano. (2023) 17:10996–1013. doi: 10.1021/acsnano.3c03252 37227087

[53] Zhou K. Strategies to promote abundance of akkermansia muciniphila, an emerging probiotics in the gut, evidence from dietary intervention studies - ScienceDirect. J Funct Foods. (2017) 33:194–201. doi: 10.1016/j.jff.2017.03.045 30416539 PMC6223323

[54] Ghotaslou R. The metabolic, protective, and immune functions of akkermansia muciniphila. Microbiol Res. (2023) 266:127245. doi: 10.1016/j.micres.2022.127245 36347103

[55] Mims BM Enriquez J dos Santos AP Jones-Hall Y Dowd S Furr KL. Antibiotic administration exacerbates acute graft vs. host disease-induced bone marrow and spleen damage in lymphopenic mice. PloS One. (2021) 16:e0254845. doi:10.1371/journal.pone.0254845 34358240 PMC8346256

[56] Qu S Zheng YH Huang YC Feng YC Xu KY Zhang W. Excessive consumption of mucin by over-colonized akkermansia muciniphila promotes intestinal barrier damage during Malignant intestinal environment. Front Microbiol. (2023) 14. doi: 10.3389/fmicb.2023.1111911 PMC10018180 36937258

[57] Sonoyama K Fujiwara R Takemura N Ogasawara T Watanabe J Ito H. Response of gut microbiota to fasting and hibernation in syrian hamsters. Appl Environ Microbiol. (2009) 75:6451–6. doi: 10.1128/AEM.00692-09 PMC2765128 19700553

[58] Elizabeth KC Jeffrey IG Stephen MS Rob K. Postprandial remodeling of the gut microbiota in burmese pythons. ISME J. (2010) 4:1375–85. doi:10.1038/ismej.2010.71 PMC3923499 20520652

[59] Rieg T Xue J Stevens M Thomas L White JR Rieg JAD. Intravenous ferric carboxymaltose and ferric derisomaltose alter the intestinal microbiome in female iron-deficient anemic mice. Biosci Rep. (2023) 43:BSR20231217. doi: 10.1042/BSR20231217 37671923 PMC10520285

[60] Zhou S Yang JY Pan YN Feng XY Hu H Ma SC. Pu’ er raw tea extract alleviates DSS-induced colitis in mice by restoring intestinal barrier function and maintaining gut microbiota homeostasis. Food Biosci. (2023) 53:102750. doi: 10.1016/j.fbio.2023.102750

[61] Zhang K Qin X Qiu JH Sun T Qu K Din AU. Desulfovibrio desulfuricans aggravates atherosclerosis by enhancing intestinal permeability and endothelial TLR4/NF-κB pathway in Apoe–/– mice. Genes Dis. (2023) 10:239–53. doi: 10.1016/j.gendis.2021.09.007 PMC10066333 37013030

[62] Gilmour CC Soren AB Gionfriddo CM Podar M Wall JD Brown SD. Pseudodesulfovibrio mercurii sp. nov., a Mercury-methylating bacterium isolated from sediment. Int J Syst Evol Microbiol. (2021) 71:004697. doi:10.1099/ijsem.0.004697 33570484

[63] Larzábal M Da Silva WM Multani A Vagnoni LE Moore DP Marin MS. Early immune innate hallmarks and microbiome changes across the gut during escherichia coli O157: H7 infection in cattle. Sci Rep. (2020) 10:21535. doi: 10.1038/s41598-020-78752-x 33299023 PMC7726576

[64] Chen L Wilson JE Koenigsknecht MJ Chou WC Montgomery SA Truax AD. NLRP12 attenuates colon inflammation by maintaining colonic microbial diversity and promoting protective commensal bacterial growth. Nat Immunol. (2017) 18:541–51. doi: 10.1038/ni.3690 PMC5395345 28288099

[65] Li YX Liu C Luo J Zeng Y Meng XL Wang SH. Ershiwuwei lvxue pill alleviates rheumatoid arthritis by different pathways and produces changes in the gut microbiota. Phytomedicine. (2022) 107:154462. doi: 10.1016/j.phymed.2022.154462 36162242

[66] Liu PY Xia D McGonigle K Carroll AB Chiango J Scavello H. Immune-mediated hematological disease in dogs is associated with alterations of the fecal microbiota: a pilot study. Anim Microbiome. (2023) 5:46. doi: 10.1186/s42523-023-00268-2 37770990 PMC10540429

[67] Zhang KK Yang JZ Chen LJ He JT Qu D Zhang Z. Gut microbiota participates in polystyrene microplastics-induced hepatic injuries by modulating the gut–liver axis. ACS Nano. (2023) 17:15125–45. doi: 10.1021/acsnano.3c04449 37486121

[68] Chen YC Ye LF Zhu J Chen L Chen H Sun YH. Disrupted tuzzerella abundance and impaired l-glutamine levels induce treg accumulation in ovarian endometriosis: a comprehensive multi-omics analysis. Metabolomics. (2024) 20:32. doi: 10.1007/s11306-023-02072-0 38424274 PMC10904428

[69] Qian L Tian SS Jiang S Tang Y Han T. DHA-enriched phosphatidylcholine from clupea harengus roes regulates the gut–liver axis to ameliorate high-fat diet-induced non-alcoholic fatty liver disease. Food Funct. (2022) 13:11555–67. doi: 10.1039/D2FO02672D 36263717

[70] Tian JS Zhang X Liu H Xiang H Xing J Zhang LZ. The hematinic effect of colla corii asini (ejiao) using 1H-NMR metabolomics coupled with correlation analysis in APH-induced anemic rats. RSC Adv. (2017) 7:8952–62. doi: 10.1039/C6RA26899D

[71] Campos-Ferraz PL Bozza T Nicastro H Lancha AH. Distinct effects of leucine or a mixture of the branched-chain amino acids (leucine, isoleucine, and valine) supplementation on resistance to fatigue, and muscle and liver-glycogen degradation, in trained rats. Nutr. (2013) 29:1388–94. doi: 10.1016/j.nut.2013.05.003 24103516

[72] Hua YL Yao WL Ji P Wei YM. Integrated metabonomic–proteomic studies on blood enrichment effects of angelica sinensis on a blood deficiency mice model. Pharm Biol. (2017) 55:853–63. doi: 10.1080/13880209.2017.1281969 PMC6130503 28140733

[73] Li PL Sun HG Hua YL Ji P Zhang L Li JX. Metabolomics study of hematopoietic function of *angelica sinensis* on blood deficiency mice model. J Ethnopharmacol. (2015) 166:261–9. doi: 10.1016/j.jep.2015.03.010 25797116

[74] Shao YY Qi WW Zhang XM Ran NY Liu CY Fu R. Plasma metabolomic and intestinal microbial analyses of patients with severe aplastic anemia. Front Cell Dev Biol. (2021) 9. doi: 10.3389/fcell.2021.669887 PMC8419359 34497802

[75] Morgan MY Milsom JP Sherlock S. Plasma ratio of valine, leucine and isoleucine to phenylalanine and tyrosine in liver disease. Gut. (1978) 19:1068–73. doi: 10.1136/gut.19.11.1068 PMC1412250 730076

[76] Li SJ Lin H Qu C Tang YP Shen J Li WX. Urine and plasma metabonomics coupled with UHPLC-QTOF/MS and multivariate data analysis on potential biomarkers in anemia and hematinic effects of herb pair gui-hong. J Ethnopharmacol. (2015) 170:175–83. doi: 10.1016/j.jep.2015.05.019 25985767

[77] Brigo N Neumaier E Pfeifhofer-Obermair C Grubwieser P Engl S Berger S. Timing of interleukin-4 stimulation of macrophages determines their anti-microbial activity during infection with salmonella enterica serovar typhimurium. Cells. (2023) 12:1164. doi: 10.3390/cells12081164 37190073 PMC10137269

[78] Bellissimo MP Roberts JL Jones DP Liu KH Taibl KR Uppal K. Metabolomic associations with serum bone turnover markers. Nutr. (2020) 12:3161. doi: 10.3390/nu12103161 PMC7602719 33081124

[79] Guo XF Zhang X Li M Peng YL Wang Z Liu J. Preliminary screening of biomarkers and drug candidates in a mouse model of β-thalassemia based on quasi-targeted metabolomics, *Front* . Physiol. (2024) 15:1452558. doi:10.3389/fphys.2024.1452558 PMC11377281 39247159

[80] Musharraf SG Iqbal A Ansari SH Parveen S Khan IA Siddiqui AJ. β-thalassemia patients revealed a significant change of untargeted metabolites in comparison to healthy individuals. Sci Rep. (2017) 7:42249. doi: 10.1038/srep42249 28198811 PMC5304209

[81] He Y Jiang HJ Du KQ Wang SJ Li MM Ma C. Exploring the mechanism of taohong siwu decoction on the treatment of blood deficiency and blood stasis syndrome by gut microbiota combined with metabolomics. Chin Med. (2023) 18:44. doi: 10.1186/s13020-023-00734-8 37088809 PMC10122815

[82] Bartlett AL Romick-Rosendale L Nelson A Abdullah S Luebbering N Bartlett J. Tryptophan metabolism is dysregulated in individuals with fanconi anemia. Blood Adv. (2021) 5:250–61. doi: 10.1182/bloodadvances.2020002794 PMC7805342 33570643

[83] Sriwichaiin S Thiennimitr P Thonusin C Sarichai P Buddhasiri S Kumfu S. Deferiprone has less benefits on gut microbiota and metabolites in high iron-diet induced iron overload thalassemic mice than in iron overload wild-type mice: a preclinical study. Life Sci. (2022) 307:120871. doi: 10.1016/j.lfs.2022.120871 35952729

[84] Tuohongerbieke A Wang HY Wu JH Wang ZQ Dong TX Huang YM. Xiao cheng qi decoction, an ancient chinese herbal mixture, relieves loperamide-induced slow-transit constipation in mice: an action mediated by gut microbiota. Pharm. (2024) 17:153. doi: 10.3390/ph17020153 PMC10892578 38399368

[85] Zhang JQ Zhao QY Qin YC Si W Zhang HY Zhang JM. The effect of epimedium isopentenyl flavonoids on the broiler gut health using microbiomic and metabolomic analyses. Int J Mol Sci. (2023) 24:7646. doi: 10.3390/ijms24087646 37108810 PMC10141048

[86] Niu C Hu XL Yuan ZW Xiao Y Ji P Wei YM. Pulsatilla decoction improves DSS-induced colitis via modulation of fecal-bacteria-related short-chain fatty acids and intestinal barrier integrity. J Ethnopharmacol. (2023) 300:115741. doi: 10.1016/j.jep.2022.115741 36162543

[87] Luo J Deng YJ Ding Y Zhang Y Tan M Xu GN. Investigation into actions of xiebai and zengye decoction on cough sensitivity, airway inflammation and gut microbiota in the rat model of post-infectious cough. Heliyon. (2023) 9:e22782. doi: 10.1016/j.heliyon.2023.e22782 38094068 PMC10716551

[88] Zhu XF Zhang XX Shen JY Zheng SS Li HZ Han B. Gut microbiota-dependent modulation of pre-metastatic niches by jianpi yangzheng decoction in the prevention of lung metastasis of gastric cancer. Phytomedicine. (2024) 128:155413. doi: 10.1016/j.phymed.2024.155413 38513377

[89] Zhang F Yung KKL KongYeung C. Effects of common prebiotics on iron status and production of colonic short-chain fatty acids in anemic rats. Food Sci Hum Wellness. (2021) 10:327–34. doi: 10.1016/j.fshw.2021.02.024

[90] Zhang Y Ye T Gong S Hong Z Zhou X Liu H. RNA-sequencing based bone marrow cell transcriptome analysis reveals the potential mechanisms of E’jiao against blood-deficiency in mice. Biomedicine RNA-sequencing based Bone marrowPharmacotherapy. (2019) 118:109291. doi: 10.1016/j.biopha.2019.109291 31401395

[91] Gaspar BL Sharma P Das R. Anemia in Malignancies: pathogenetic and diagnostic considerations. Hematology. (2015) 20:18–25. doi: 10.1179/1607845414Y.0000000161 24666207

[92] Nakamura-Ishizu A Takubo K Kobayashi H Suzuki-Inoue K Suda T. CLEC-2 in megakaryocytes is critical for maintenance of hematopoietic stem cells in the bone marrow. J Exp Med. (2015) 212:2133–46. doi: 10.1084/jem.20150057 PMC4647260 26552707

[93] Sakai H Chen Y Itokawa T Yu KL Zhu ML Insogna K. Activated c-fms recruits vav and rac during CSF-1-induced cytoskeletal remodeling and spreading in osteoclasts. Bone. (2006) 39:1290–301. doi: 10.1016/j.bone.2006.06.012 16950670

[94] Cai ZS Deng X Jia J Wang D Yuan GY. Ectodysplasin a/ectodysplasin a receptor system and their roles in multiple diseases. Front Physiol. (2021) 12. doi: 10.3389/fphys.2021.788411 PMC8685516 34938205

[95] Chen KH Wang P Chen JR Ying YL Chen Y Gilson E. Loss of atm in zebrafish as a model of ataxia–telangiectasia syndrome. Biomedicines. (2022) 10:392. doi: 10.3390/biomedicines10020392 35203601 PMC8962326

[96] Anjos-Afonso F Loizou J Bonnet D Behrens A. Perturbed hematopoiesis In mice lacking ATMIN (an ATM co-factor). Blood. (2013) 122:2412. doi: 10.1182/blood.V122.21.2412.2412 27581360 PMC5147016

[97] Maekawa S Pulpipat T Wang PC Chen SC. Transcriptome analysis of immune- and iron-related genes after francisella noatunensis subsp. orientalis infection in nile tilapia (oreochromis niloticus). Fish Shellfish Immunol. (2021) 111:36–48. doi: 10.1016/j.fsi.2020.11.009 33444737

[98] Tenen DG. Disruption of differentiation in human cancer: AML shows the way. Nat Rev Cancer. (2003) 3:89–101. doi: 10.1038/nrc989 12563308

[99] Walter K Bonifer C Tagoh H. Stem cell–specific epigenetic priming and B cell–specific transcriptional activation at the mouse Cd19 locus. Blood. (2008) 112:1673–82. doi: 10.1182/blood-2008-02-142786 18552207

[100] O’Keefe TL Williams GT Davies SL Neuberger MS. Hyperresponsive B cells in CD22-deficient mice. Sci. (1996) 274:798–801. doi: 10.1126/science.274.5288.798 8864124

[101] Uckun FM Goodman P Ma H Dibirdik I Qazi S. Intronic CD22 gene mutations as a pathogenic mechanism of human B-precursor leukemia. Blood. (2010) 116:149. doi: 10.1182/blood.V116.21.149.149 PMC2947921 20841423

[102] Huang FY Zhang JL Zhou H Qu TY Wang Y Jiang KX. B cell subsets contribute to myocardial protection by inducing neutrophil apoptosis after ischemia and reperfusion. JCI Insight. (2024) 9:e167201. doi: 10.1172/jci.insight.167201 38290007 PMC10967377

[103] Beekman JM Verhagen LP Geijsen N Coffer PJ. Regulation of myelopoiesis through syntenin-mediated modulation of IL-5 receptor output. Blood. (2009) 114:3917–27. doi: 10.1182/blood-2009-03-208850 19654410

[104] Mansour A Abou-Ezzi G Sitnicka E Jacobsen SEW Wakkach A Blin-Wakkach C. Osteoclasts promote the formation of hematopoietic stem cell niches in the bone marrow. J Exp Med. (2012) 209:537–49. doi: 10.1084/jem.20110994 PMC3302238 22351931

[105] Wang QS Shi QQ Wang ZQ Lu JW Hou J. Integrating plasma proteomes with genome-wide association data for causal protein identification in multiple myeloma. BMC Med. (2023) 21:377. doi: 10.1186/s12916-023-03086-0 37775746 PMC10542236

[106] Aygun D Koker MY Nepesov S Koker N van Leeuwen K de Boer M. Genetic characteristics, infectious, and noninfectious manifestations of 32 patients with chronic granulomatous disease. Int Arch Allergy Immunol. (2020) 181:540–50. doi: 10.1159/000507366 32512560

[107] Peng HY Zou Y. The leukocyte immunoglobulin-like receptor gp49B1, coexpressed with c-kit, modulates hematopoiesis and B cell leukemia development. Biochem Biophys Res Commun. (2021) 565:72–8. doi: 10.1016/j.bbrc.2021.05.090 34098314

[108] Walkley CR. Erythropoiesis, anemia and the bone marrow microenvironment. Int J Hematol. (2011) 93:10–3. doi: 10.1007/s12185-010-0759-6 21222184

[109] Miyajima A Kinoshita T Tanaka M Kamiya A Mukouyama Y Hara T. Role of oncostatin M in hematopoiesis and liver development. Cytokine Growth Factor Rev. (2000) 11:177–83. doi: 10.1016/S1359-6101(00)00003-4 10817961

[110] Tanaka M Hirabayashi Y Sekiguchi T Inoue T Katsuki M Miyajima A. Targeted disruption of oncostatin M receptor results in altered hematopoiesis. Blood. (2003) 102:3154–62. doi: 10.1182/blood-2003-02-0367 12855584

[111] Sathyanarayana P Menon MP Bogacheva O Bogachev O Niss K Kapelle WS. Erythropoietin modulation of podocalyxin and a proposed erythroblast niche. Blood. (2007) 110:509–18. doi: 10.1182/blood-2006-11-056465 PMC1924484 17403918

[112] Walker EC McGregor NE Poulton IJ Solano M Pompolo S Fernandes TJ. Oncostatin M promotes bone formation independently of resorption when signaling through leukemia inhibitory factor receptor in mice. J Clin Invest. (2010) 120:582–92. doi: 10.1172/JCI40568 PMC2810087 20051625

[113] Qiao YM Hu H Zhao YY Jin M Yang D Yin J. Benzene induces spleen injury through the B cell receptor signaling pathway. Ecotoxicol Environ Saf. (2023) 257:114924. doi: 10.1016/j.ecoenv.2023.114924 37080132

[114] Yin Z Ou RM Zhu YM Liu Z Huang J Zhong Q. Coniferyl ferulate alleviate xylene-caused hematopoietic stem and progenitor cell toxicity by Mgst2. Front Pharmacol. (2024) 15. doi: 10.3389/fphar.2024.1334445 PMC10957570 38523643

[115] Wu XJ Jiao JC Xia YF Yan XT Liu ZH Cao YY. Case report: a chinese patient with glutathione synthetase deficiency and a novel glutathione synthase mutation. Front Pediatr. (2023) 11. doi: 10.3389/fped.2023.1212405 PMC10413980 37576147

[116] Altamura S Vegi NM Hoppe PS Schroeder T Aichler M Walch A. Glutathione peroxidase 4 and vitamin E control reticulocyte maturation, stress erythropoiesis and iron homeostasis. Hematol. (2020) 105:937–50. doi: 10.3324/haematol.2018.212977 PMC7109755 31248967

[117] Lee S Kwon HC Kim SH Oh SY Lee JH Lee YS. Identification of genes underlying different methylation profiles in refractory anemia with excess blast and refractory cytopenia with multilineage dysplasia in myelodysplastic syndrome. Korean J Hematol. (2012) 47:186–93. doi: 10.5045/kjh.2012.47.3.186 PMC3464335 23071473

[118] Ito M Tanaka T Ishii T Wakashima T Fukui K Nangaku M. Prolyl hydroxylase inhibition protects the kidneys from ischemia via upregulation of glycogen storage. Kidney Int. (2020) 97:687–701. doi: 10.1016/j.kint.2019.10.020 32033782

[119] Sun NC Wang ZL Jiang HH Wang BY Du KH Huang CH. Angelica sinensis polysaccharides promote extramedullary stress erythropoiesis via ameliorating splenic glycolysis and EPO/STAT5 signaling-regulated macrophages. J Mol Histol. (2024) 55:661–73. doi: 10.21203/rs.3.rs-4180160/v1 38969952

[120] Castoldi GL Merker H. Nachweis und bedeutung histochemisch darstellbarer cholin-dehydrogenase in menschlichen blut- und knochenmarkzellen. Klin Wochenschr. (1965) 43:368–75. doi: 10.1007/BF01484657 14291094

