# Supplementary material for: Hematopoietic effects of Fufang E’jiao Jiang revealed by microbiome, metabolome and transcriptome analyses: a multi-omics strategy
Source: Front Immunol. 2025 Jun 12;16:1561477. doi: 10.3389/fimmu.2025.1561477 (PMC12197926; doi:10.3389/fimmu.2025.1561477)
Supplement: Supplementary file 1 [file DataSheet1.docx]

Supplementary Material

**
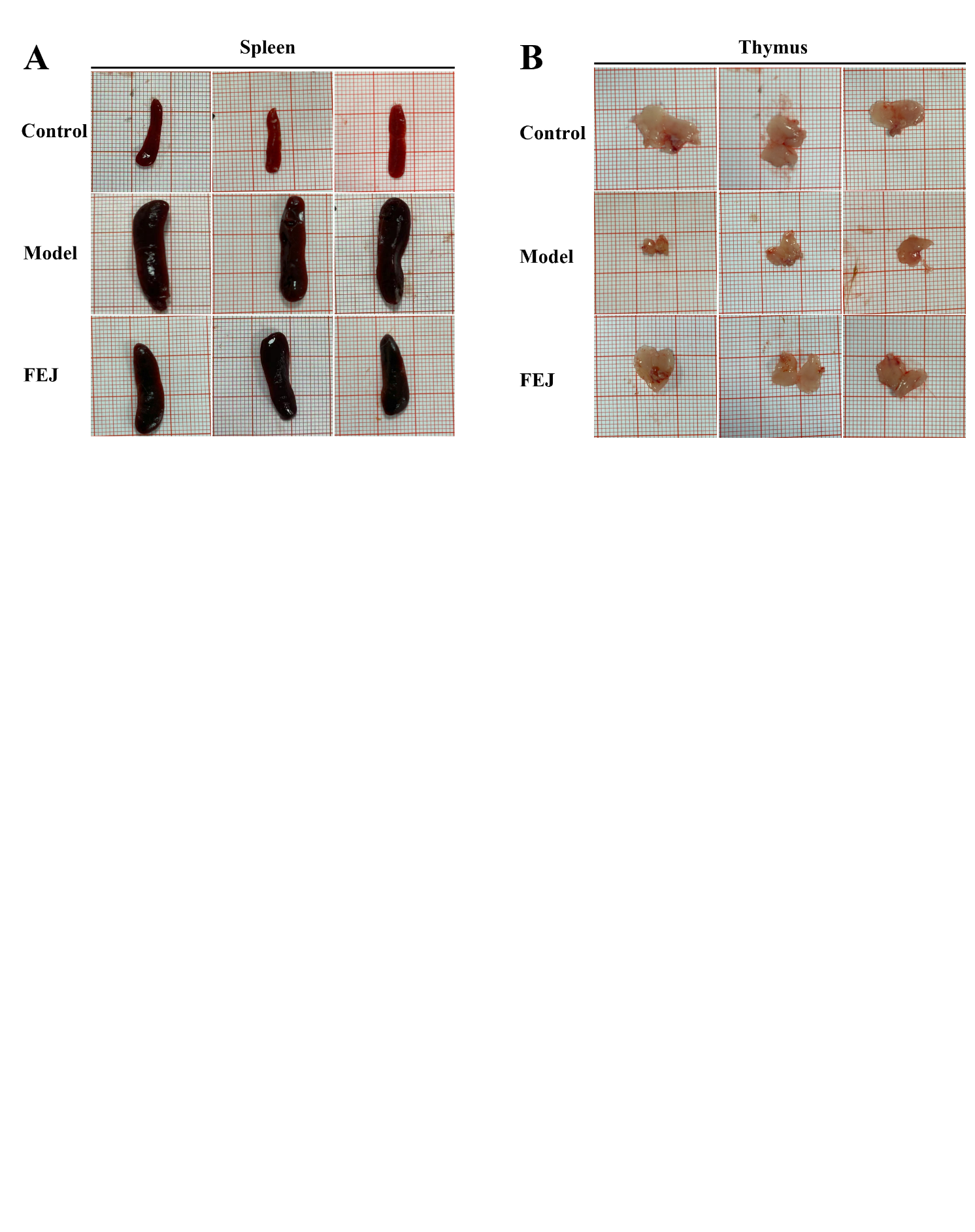
Supplementary Figure 1.** Representative images of spleen (A) and thymus (B) from mice in each group under stereoscope.
